# Patterns of variation in DNA segments upstream of transcription start sites

**DOI:** 10.1002/humu.20463

**Published:** 2007-05

**Authors:** Damian Labuda, Catherine Labbé, Sylvie Langlois, Jean-Francois Lefebvre, Virginie Freytag, Claudia Moreau, Jakub Sawicki, Patrick Beaulieu, Tomi Pastinen, Thomas J Hudson, Daniel Sinnett

**Affiliations:** 1 Centre de Recherche, Hôpital Sainte-JustineMontréal, Quebec, Canada; 2 Département de Pédiatrie, Université de MontréalMontréal, Quebec, Canada; 3 McGill University and Genome Quebec Innovation CenterMontreal, Quebec, Canada

**Keywords:** DNA diversity, promoter regions, haplotypes, selective sweeps, human populations

## Abstract

It is likely that evolutionary differences among species are driven by sequence changes in regulatory regions. Likewise, polymorphisms in the promoter regions may be responsible for interindividual differences at the level of populations. We present an unbiased survey of genetic variation in 2-kb segments upstream of the transcription start sites of 28 protein-coding genes, characterized in five population groups of different geographic origin. On average, we found 9.1 polymorphisms and 8.8 haplotypes per segment with corresponding nucleotide and haplotype diversities of 0.082% and 58%, respectively. We characterized these segments through different summary statistics, Hardy-Weinberg equilibria fixation index (Fst) estimates, and neutrality tests, as well as by analyzing the distributions of haplotype allelic classes, introduced here to assess the departure from neutrality and examined by coalescent simulations under a simple population model, assuming recombinations or different demography. Our results suggest that genetic diversity in some of these regions could have been shaped by purifying selection and driven by adaptive changes in the other, thus explaining the relatively large variance in the corresponding genetic diversity indices loci. However, some of these effects could be also due to linkage with surrounding sequences, and the neutralists' explanations cannot be ruled out given uncertainty in the underlying demographic histories and the possibility of random effects due to the small size of the studied segments. Hum Mutat 28(5), 441–450, 2007. © 2007 Wiley-Liss, Inc.

## INTRODUCTION

Patterns of DNA diversity in the human genome result from the stochastic nature of mutations and recombinations. They are further shaped by random genetic drift, by demographic history, and by natural selection. To understand the distinct contributions of the underlying genetic and evolutionary phenomena, we have to examine the genomic variation in individuals and populations from different geographic areas. So far, considerable effort has gone into the identification of polymorphisms which could be used as markers in linkage and association studies. Fewer studies have focused on DNA variability in particular genomic segments, and even fewer have investigated variation beyond two or three population groups. Likewise, much less attention has been given to DNAvariants that could be classified as regulatory, in spite of their importance for understanding species evolution [King and Wilson, 1975], functional components of the genome [Carroll, 2005], and ultimately as phenotypic diversity and complex traits [Pastinen and Hudson, 2004]. On the other hand, there is an inherent difficulty in defining DNA segments and their variants that can be qualified as regulatory—in contrast, for example, to easily qualifiable alterations in the protein-coding sequences. In order to describe genetic diversity in cis-regulatory regions and, in particular, DNA variation involved in the control of transcription of the protein-coding genes, we used an operational definition of the promoter region as a 2-kb segment upstream of the transcription start site [Kim et al., 2005]. Previously, we described DNA polymorphisms in 197 such regions surveyed for the presence of variants in a sample of 40 individuals representing five population groups distributed worldwide [Sinnett et al., 2006]. These loci were primarily chosen as candidate genes for their possible involvement in cancer [Belanger et al., 2005], drug response, inflammation, and/or displaying allelic imbalance [Pastinen et al., 2004]. In this article, we present a detailed characterization of genetic diversity in upstream regions of a subset of 28 of these genes, and we describe the corresponding allelic and haplotypic frequencies as well as their geographic distributions. The resulting diversity patterns portray different landscapes of genomic variability, suggesting as well that natural selection could have influenced the evolution of some of these segments.

## MATERIALS AND METHODS

### Supplementary Data

Supplementary Tables S1, S2, S3, and S4, and Supplementary Figures S1, S2, S3, and S4 are available online at http://www.interscience.wiley.com/jpages/1059-7794/suppmat and present data essential to this analysis.

### DNA Samples

DNA samples were obtained from the Coriell Institute for Medical Research (Camden, NJ) or were isolated from peripheral blood donated on an anonymous basis by consenting adults according to a protocol approved by the Institutional Review Board (at the Hôpital Sainte-Justine). A total of 40 unrelated samples representing five population groups were used to search for DNA variants that were subsequently confirmed by genotyping of the same samples using allele-specific oligonucleotide (ASO) hybridization. Sample groups were as follows: 1) sub-Saharan Africans including Biaka and M'Buti Pygmies (NA10469-71, Na10492) and African-Americans (NA17101, NA17108-10); 2) Americans of Amerindian descent from Brazil, Guyana, and Venezuela (NA17311-12, NA17314-20); 3) Europeans, represented by French-Canadians and Central Europeans (from Bulgaria, Germany, and Poland); 4) East and Southeast Asians from China and Indochina (Cambodia, Laos, and Vietnam); and 5) Southwestern Asians, represented by samples obtained from individuals of Jewish and Arab descent. A total of 40 additional samples from which genotypes were also determined were only compared in the calculation of Hardy-Weinberg equilibria (HWE) and fixation index (Fst) estimates. They included: 1) Africans South of the Sahara (NA17341-48); 2) Americans of Amerindian descent from the Andes (NA17303-10); 3) Hungarians (NA15199-202) and Russians (NA13838, NA13849, NA13852, NA13876); 4) Chinese (NA16654, NA16688-89, and NA17014-18); and 5) Indo-Pakistanis (NA17021-29). DNA samples of three great apes–common chimpanzees, gorillas, and orangutans—from Granby Zoo (Institut de la statistique du Québec) were included in ASO hybridization as well, to infer the ancestral allele at every polymorphic site.

### Polymorphisms and Genotyping

Polymorphisms were detected by dHPLC (WAVE System of Transgenomic; Transition Technologies Inc., Toronto, Ontario, Canada) and sequencing as described [Sinnett et al., 2006]. Briefly, typically seven amplicons of ∼300 bp were designed (Primer 3 Software [Rozen and Skaletsky, 2000]) to cover the 2-kb segment upstream of the transcription start site. DNA fragments were amplified by standard PCR, using 10 ng of genomic template, and the products were analyzed by dHPLC at a minimum of two different temperatures. The purported heteroduplexes were subsequently sequenced (Applied Biosystems 3730 xl DNA Analyzer; Applied Biosystems, Foster City, CA) to identify the underlying variants. Allelic states of substitution and small insertion/deletion polymorphisms in the human and in the great apes samples were determined by dynamic ASO hybridization as described [Bourgeois and Labuda, 2004], using the ASO probes listed in Supplementary Table S2. To avoid transcription errors, the genotypes were read and entered into our database twice by two independent individuals, and only concordant readings were accepted.

### Statistical Analyses

Haplotypes for each ∼2-kb segment were derived from the corresponding genotypes using the PHASE program v. 2.0 (www.stat.washington.edu/stephens/software.html) [Stephens et al., 2001]. Haplotype networks were drawn manually, assisted by Phylogenetic Network Analysis Software (www.fluxus engineering.com) [Bandelt et al., 1999]. We used ARLEQUIN software, v. 2.0 (http://lgb.unige.ch/arlequin) [Schneider et al., 2000], DnaSP package v. 4.0 (www.ub.es/dnasp) [Rozas et al., 2003], and H-test software (www.genetics.wustl.edu/jflab/htest.html) [Fay and Wu, 2000], to compute population parameters, population variance Fst, Hardy-Weinberg equilibria, summary statistics, and to carry out neutrality tests.

Tajima's *D* test statistics [Tajima, 1989] considers the difference between θ_S_ and θ_π_, normalized by the expected standard deviation of this difference. The first estimator, θ_S_=S/Σ^n−1^_*i*_ 1/i, where n corresponds to the number of sampled chromosomes [Watterson, 1975], is only influenced by the number of segregating sites, S. The second estimator, Yp, representing the mean number of pairwise differences between individual sequences [Tajima, 1989], corresponds to the product of nucleotide diversity and the sequence length *L* such that θ_π_. The number of sites, S1, e., the number of sites with a derived allele observed only once (*i*)=*l*), provides the estimator θ_F_=S_1_ [Fu and Li, 1993]. YF is used in conjunction with either θ_S_ or θ_π_ to calculate test statistics *D*_Fu&Li_ and F, respectively, as well as related statistics D^*^ and F^*^ [Fu and Li, 1993]. The estimator θ_H_, by Fay and Wu, corresponds to the sum of twice the squared frequency of the derived alleles [Fay and Wu, 2000]. The corresponding *H*-statistics comparing θ_π_ with θ_H_ are particularly sensitive to selection sweeps and the effect of genetic hitchhiking [Maynard Smith and Haigh, 1974], whereby a positively selected variant simultaneously drives up the population frequency of its neighboring tightly linked alleles. The test statistics *F*_s_ Fu, 1997] examine the difference in the number of the observed haplotypes with those expected given θ_π_, thus confronting the latter with θ_*k*_. Tests by Ewens-Watterson and Chakraborty [Chakraborty, 1990; Ewens, 1972; Watterson, 1978] based on the infinite allele (haplotype) model can be considered to some extent complementary; they confront θ_*k*_, estimated from the number of haplotypes with θ_hom_, estimated from the haplotype homozygosity, hom=1−G. Evaluating the significance of the Ewens-Watterson test, Arlequin implements the protocol described by Watterson [1978] and that by Slatkin [1994]. All neutrality tests were carried out using data obtained with an initially ascertained sample of 80 chromosomes, except in the Fay and Wu test, where an extended sample of 80 genotyped individuals was used. The Hudson-Kreitman-Aguadé (HKA) test [Hudson et al. 1987]—considering the number of segregating sites, *S*, as well as nucleotide diversity, π, as a measure of locus diversity—was executed as previously described [Jaruzelska et al., 1999]. The reported Fsts and the results of the HWE test were obtained using genotypes of the extended sample of 80 individuals. Correction for multiple testing (neutrality two-tailed and HWE tests) was carried out according to Storey [2002] using a false discovery rate of 10% (http://faculty.washington.edu/jstorey/qvalue/index.html).

Divergence, *d*, between human and chimpanzee sequences was estimated by counting differences between the human sequence (Supplementary Table S1) and the chimpanzee sequence (November 2003 Assembly UCSC Browser [http://genome.ucsc. edu-cgi-bin/hgGateway]), initially at the overlapping length with the analyzed 2-kb human segment and subsequently extended to about 12 kb by adding 5 kb on each side to decrease variance in the d estimate. Both estimates largely agreed, and the latter is reported (Table 1). Prism v. 4.03 (GraphPad Software Inc., San Diego, CA), Excel (Microsoft, Redmond, WA), and Statistica v. 7.1 (StatSoft Inc., Tulsa, OK) were used to evaluate distributions and in correlation analyses.

**TABLE 1:** Diversity Indices and Different Estimators of θ (for a Sample of 80 InitiallyAscertained Chromosomes)

														F_st_(%)^c^	
														
	*k*	*S*	*G*	*L*	*d*(%)	θ_*S*_	θ_π_	θ_*H*_	θ_*F*_	θ_*hom*_	θ_k_	*M*^a^	Four gamete test^b^	Total sample	Non African sample
BTN3A2	9	14	0.57	2032	1.09	2.84	2.20	5.62	5	0.98	2.42	5		ns	ns
CAT	8	7	0.74	2092	1.81	1.41	1.65	0.69	1	2.17	2.02	1	(1)	7.9***	8.5***
CCNDI	11	13	0.62	1606	1.28	2.62	1.43	0.99	3	1.258	3.23	1	(1)	ns	ns
CCNEI	3	2	0.10	844	1.25	0.40	0.10	0.00	1	0.08	0.46	0		ns	ns
CDC25A	7	7	0.34	1664	1.69	1.41	0.38	0.01	3	0.37	1.66	0		7.5**	8.2***
CDKNIA	13	11	0.76	1597	1.53	2.22	3.18	2.58	0	2.42	4.16	2	+	3.7***	ns
CDKNIB	11	10	0.78	2012	1.29	2.02	1.60	0.42	2	2.74	3.23	1/0		7.5***	6.9**
CDKN2A	7	8	0.35	2069	0.90	1.61	0.58	0.03	2	0.41	1.66	0		7.1***	6.7***
CX3CRI	17	16	0.86	1987	1.25	3.23	4.03	6.07	2	5.01	6.32	6	+	11.4***	7.8***
E2F1	5	4	0.43	1925	1.09	0.81	0.48	0.08	2	0.55	1.01	0		ns	ns
FEN1	6	5	0.55	1992	0.86	1.01	0.61	0.30	3	0.91	1.32	0		18.7***	9.8**
FGB	11	13	0.80	1951	0.91	2.62	2.06	1.72	5	3.09	3.23	1		11.4***	9.9***
GPX2	17	16	0.74	2077	0.90	3.23	2.27	2.98	6	2.17	6.32	2	+	15.1***	18.5***
GPX3	10	11	0.64	2157	1.05	2.25	2.61	7.61	2	1.31	2.89	6	+	7.4***	ns
GSS	9	9	0.73	1931	1.26	1.82	1.59	2.45	3	2.11	2.40	2		3.4**	ns
GSTM3	7	6	0.70	1951	1.09	1.21	1.38	3.99	2	1.82	1.66	3	(1)	9.3***	6.4***
GSTM4	9	10	0.73	1780	1.11	2.02	2.14	1.59	2	2.05	2.40	1		ns	ns
GSTPI	12	13	0.71	2060	1.38	2.62	4.11	5.10	0	1.89	3.68	5	+	5.7***	2.1*
HDACI	11	12	0.67	2029	1.55	2.42	1.03	2.39	5	1.52	3.23	2		18.6***	ns
HTR2A	13	18	0.71	2053	1.58	3.63	2.39	6.84	6	1.83	4.16	5	+	1.9*	ns
IL1A	4	3	0.67	2008	1.26	0.60	0.99	0.31	0	1.58	0.73	0		13.5***	4.7*
MICA	17	15	0.87	2164	2.12	3.04	1.61	0.26	5	5.38	6.41	0	(1)	3.5*	3.9*
RBI	6	4	0.44	1963	0.98	1.01	0.53	0.06	1	0.59	1.32	0		3.9*	ns
SKP2	3	2	0.10	1934	1.00	0.40	0.10	0.00	0	0.08	0.46	0		3.7*	ns
SMAD3	5	5	0.72	1366	1.53	1.01	1.32	0.32	0	1.94	1.01	0		ns	ns
SMAD4	2	2	0.03	1725	1.03	0.40	0.05	0.00	2	0.02	0.22	0		7.7***	ns
TFDPI	4	8	0.28	939	1.92	1.62	0.83	0.05	0	0.29	0.73	0		10.6***	7.0***
TGFBI	9	9	0.62	2012	1.31	1.82	2.18	1.74	4	1.25	2.42	2		ns	ns

a Number of mutational steps from ancestral to the observed major haplotype (i.e. allelic class of the most frequent haplotype)

b (1) indicates the presence of only one recombinant haplotype (see Supplementary Fig.1).

c Level of significance as from ARLEQUIN; F_ST_values were calculated for an extended sample of 80 genotyped individuals.

*** *P*<0.001

** *P*<0.01

*** *P*<0.001

ns,not significant.

Coalescent simulations [Hudson, 1990] were performed under a selectively neutral model using the Cosi program of Schaffner et al. [2005] (www.broad.mit.edu/personal/sfs/cosi). We carried out these simulations using mutation rates defined by the average polymorphic site's density and N510,000 to obtain distributions of: 1) the number of haplotypes carrying distinct numbers (m50, 1, 2, etc.) of new alleles; 2) the frequencies of the ancestral haplotypes; and 3) the major haplotypes among different classes of haplotypes defined by the number of new alleles, m, that they carry. In addition to simulations under the standard model without recombination, we studied the effect of recombinations, as well as demographic expansion and/or population bottleneck as used by Akey et al. [2004].

### RESULTS

#### Diversity Data

In a group of 28 protein-coding genes (Supplementary Table S1), we characterized genetic variations in their promoter regions, defined arbitrarily as 2-kb segments upstream of the transcription start sites see Kim et al., 2005]. Transcriptional start sites were defined based on the mRNA sequence versions (REFSEQ) listed in Supplementary Table S1. DNA polymorphisms were first ascertained by dHLPC, combined with DNA sequencing, in a panel of 40 genomic samples representing individuals of sub-Saharan African, Native American, European, Middle Eastern, and Southeast/East Asian descent [Sinnett et al., 2006]. All variants reported here were genotyped and thus independently reconfirmed by ASO hybridization in the same panel of 40 individuals. We observed 254 simple polymorphisms, 243 substitutions, and 11 indels. The sequence contexts of each of the reported polymorphisms, as well as their “rs” identifiers, are provided in Supplementary Table S2, while the corresponding reconstructed haplotypes (see Materials and Methods) are listed in Supplementary Table S3. HWE was primarily tested as an additional means of checking the quality and consistency of our genotypes [e.g., Fan et al. 2002]. As a result, and after correcting for multiple testing [Storey, 2002] (see Materials and Methods), we found three polymorphisms in the CX3CR1 segment showing significant departure from the HWE (see below).

Different diversity indices and other characteristics of the analyzed loci are presented in Table 1, which also includes per segment Fst values for the whole population sample and considering only non-African groups. Geographic distribution of haplotypes is given within haplotype networks shown in Supplementary Figure S1. On average, we observed nine segregating sites (S=9.1±4.6) and a similar number of haplotypes (k=8.8±4.2) per segment (average length of 1854±329). Mean nucleotide diversity and haplotype diversity are π(%)=0.082±0.056 and G=0.58±0.23, respectively. The associated standard deviations manifest a large variance between individual loci. The extent of this variance may be due to:1) stochastic effects resulting from different genealogical histories of each of these segments; 2) the effect of selection; or 3) heterogeneity in the mutation rate among loci [Chuang and Li, 2004; Matassi et al., 1999]. However, we did not notice any systematic correlation (r^2^=0.015) between S and nucleotide divergence d, measured as a proportion of fixed sites between humans and chimpanzees (Table 1). Thus, variation in the mutation rate alone cannot explain differences between the polymorphic content in the analyzed segments. It could possibly be due to differences in the underlying genealogies, reflecting either variation in demographic history or the effect of natural selection. Examination for these effects requires the analysis of diversity indices that capture different aspects of the data.

In Table 1, we compared different estimators of the population mutation parameter Θ=4*Nµ*, where *N* denotes the effective population size and µ, the mutation rate per segment per generation. Watterson's estimator, Θ_S_ [Watterson, 1975], and Tajima's Θπ [Tajima, 1989], based on the infinite sites model, can be derived from S and π, respectively. In turn, Θ_k_ [Ewens, 1972] and Θ_hom_ [Chakraborty, 1990], estimated from *k* and G, originate in the infinite alleles model (see Materials and Methods). Note that the term “allele” is reserved here to variants of a single segregating site as in the infinite sites model and, in the infinite alleles model, should be replaced by “haplotype” (i.e., variant of the whole locus). Two additional estimators, Θ_F_ by Fu and Li [1993] and Θ_H_ by Fay and Wu [2000], require the knowledge of the derived and the ancestral state at each of the segregating sites, here obtained by genotyping chimpanzee and other ape DNAs (see Materials and Methods). Knowing the ancestral allele at each polymorphic site, we introduced an additional descriptor of locus diversity, a haplotype allelic class describing the number of mutational steps separating each haplotype from the ancestral haplotype (i.e., entirely composed of ancestral alleles). Table 1 lists haplotype allelic class M for each of the major haplotypes. The major, i.e., most frequent, haplotype in 12 of these segments is the ancestral one (i.e., M=0). In contrast, we observed M≥5 in five of these (see the correlation with corresponding Θ_Hs_). The fourgamete test indicated that recombinations contributed to haplotype diversity in 10 of the segments. Yet, as indicated in Table 1, in four of them the impact of these events was relatively small, with only one additional recombinant haplotype observed (see the corresponding haplotype networks in Supplementary Fig. S1).

We carried out neutrality tests that confront different estimates of Θ and/or summary statistics, such as the number of haplotypes or their homozygosity (Table 1). Assuming a simple model of a population at constant size, mutational equilibrium, and neutrality, these different estimates are expected to be the same or to agree, given the associated variance (see Materials and Methods). An opposite result indicates departure from this model, suggesting selection or less simple demography, i.e., effects due to population growth, bottleneck, or population structure. Table 2 lists all segments highlighted by these tests; the results that remain significant after correcting for multiple testing [Storey, 2002] using a false discovery rate of 10% are shown in bold.

**TABLE 2:** Neutrality Tests*

	Observed values				Tajima[1989]	Fu and Li[1993]	Fay and Wu [2000]^a^	Fu[1997]	Chakraborty [1990]	Ewens [1972]Watterson[1978]
							
	π(%)	S	k	hom	D(p)	*D/F(p)*	*H*(p)	k_exp_*F_s_/*(p)	k_exp_(*p*)	hom_exp_(p_w_;p_s_)
BTN3A2	0.108	14	9	0.43			−3.57	8.4	4.9	0.28
							(0.050)	ns	(0.031)	ns
CCNDI	0.090	13	11	0.38				6.4	**5.8**	0.23
								ns	**(0.014)**	(ns;0.970)
CDC25A	0.023	7	7	0.67	−**1.76**			2.7	**2.7**	0.36
					**(0.008)**			−**5.15/(0.002)**	**(0.005)**	(0.970;ns)
CDKN2A	0.027	8	7	0.65	−**1.61**			**3.5**	**2.8**	0.36
					**(0.016)**			−**3.45/(0.032)**	**(0.005)**	(0.967;ns)
GPX2	0.110	16	17	0.27				**8.9**	**8.4**	0.14
								−**6.37/(0.011)**	**(0.001)**	0.14
GPX3	0.121	11	10	0.36			−5.96	9.4	5.9	0.25
							(0.009)	ns	(0.042)	ns
GSTM3	0.068	6	7	0.33			−3.32	6.2	7.5	0.36
							(0.013)	ns	ns	ns
GSTPI	0.200	13	12	0.30		**1.88**^b^		12.9	7.7	0.21
						**(0.984)**		ns	(0.053)	(ns;0.965)
HDACI	0.051	12	11	0.34	−1.57			5.1	6.6	0.23
					(0.026)			−5.36/(0.0095)	(0.040)	(ns;0.976)
HTR2A	0.117	18	13	0.30			−4.53	9.0	**7.5**	0.19
							(0.036)	ns	**(0.019)**	(ns;0.968)
II1A	0.050	3	4	0.33				4.9	6.7	0.55
								ns	(0.946)	(ns;0.043)
MICA	0.062	15	17	0.14				6.8	15.2	0.14
								−9.84/(0.000)	ns	ns
SKP2	0.005	2	3	0.90	−1.21			1.5	1.4	0.66
					(0.041)			−2.41/(0.010)	ns	ns
SMAD4	0.003	2	2	0.97	**−1.41**	−**2.75**^b^		1.3	1.1	0.80
					**(0.012)**	**(0.048)**		ns	ns	ns
TGFBI	0.109	9	9	0.38				8.4	5.7	0.29
								ns	ns	(ns;0.982)

* Numbers in bold are the results that are significant after correction formultiple testing.

a Exceptionally, the values for the Fay and Wu [2000] test are based on a sample of 160 chromosomes.

ns, not significant.

**FIGURE 1:**
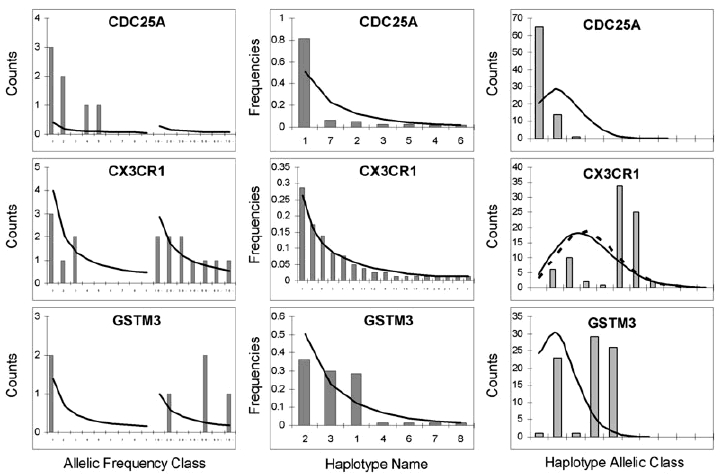
Distributions of allelic frequency classes (left panels) of frequencies of haplotypes [Middleton et al.,1993] and haplotype allelic classes (right) inCDC25A,CX3CR1, andGSRM3. Bars represent the observed values; lines represent theoretical distributions. The occupancy of allelic frequency classes corresponds to counts of sites represented by *i* new alleles in a sample of *n* chromosomes (i=1, 2, 3,…, n–1). Here, the theoretical curve (solid line) corresponds to the distribution calculated from the equation [Fan et al., 2002; Fu,1997] S_i_(i)Θ/i,using Θ/π (Table 1) as the estimator of Θ.The theoretical distribution (solid line) of haplotype frequencies expected given k observed haplotypes (Table 1) is according to Ewens [1972]. Haplotype names are arbitrary and correspond to their names in our database. In the case of haplotype allelic classes, regrouping haplotypes sharing the same number of mutations from the ancestral haplotype, their theoretical occupancy was obtained by coalescent simulation under the standard model, assuming constant population size without (solid line) and with (dotted line) recombination, at 10-fold the genomic average in the case of segments where crossovers were detected.

The testing of data in the framework of the infinite sites model can be illustrated by a histogram of allelic frequency classes that regroup sites with the same number of the derived allele, from i=1, 2, 3,…to i=n–1, where *n* is the number of chromosomes in the sample. The expected distribution is S_i_(*i*)=Θ/*i* [Fan et al., 2002; Fu, 1997] where σ S_i_=S, as illustrated in the left panels of Figure 1, where Θπ estimates (Table 1) were used to trace the theoretical curve according to the above equation. The corresponding plots for other segments than the three shown in Figure 1, either highlighted by neutrality tests or singled out by Fst statistics, can be found in Supplementary Figure S2. The histogram of allelic frequency classes in Figure 1 shows an excess of low-frequency polymorphisms in the case of CDC25A, as revealed by the negative Tajima's *D* in this segment (Table 2); it shows a good concordance between theoretical distribution and the data in the CX3XR1 segment and a marked excess of highfrequency-derived alleles in the case of GSTM3. The latter agrees with the result of the Fay and Wu test for this segment (Table 2). Middle histograms in Figure 1 illustrate the results of the haplotype-based tests. In the case of CX3CR1, as for allelic frequency classes, this plot shows an excellent fit between the theoretical distribution and the observed frequencies. In these representations illustrating the results of the neutrality test from Table 2, the CX3CR1 segment appears to conform to a simple neutral model. In contrast, in CDC25A given the number in the expected frequencies do not match with the data. There is anexcess of the observed haplotypes, given their homozygosity (1–G). This discordant distribution, in the case of the CDC25A segment, reflects significant results of haplotype-based tests, including Fu's *Fs* test, which, however, compares *k* with its estimate based on Θ_π_ rather than Θ_hom_. After correcting for multiple testing, no segment remained significant for the Ewens-Watterson test as well as for the Fay and Wu test. Furthermore, the significant results of Chakraborty's test for HTR2A and GPX2, as well as those of Fu's Fs test for the GPX2, can likely be ascribed to the effect of recombinations. The latter, causing the number of the observed haplotypes to increase faster than they would simply due to mutation alone, can render the results of the above tests falsely significant. Yet, at the same time, the presence of recombinations renders other tests, such as Tajima's or Fay and Wu's, less conservative, i.e., “more significant” [Fay and Wu, 2000]. Indeed, considering the effect of recombinations (three-to six-fold genomic average) in six segments where more than one recombinant haplotype was observed (Table 1), GPX3 stayed significant for the Fay and Wu test after the correction for multiple testing.

#### Haplotype Allelic Classes

While the left and middle histograms in Figure 1 represented the allelic and haplotype configurations of the analyzed segments, the histograms shown in the right column combine the allelic and haplotypic information in a single plot to reveal additional characteristics of the data. The haplotype allelic classes A represent all haplotypes carrying the same number of new alleles m such that, for example, A_m_=0 represents the number of the ancestral haplotypes in the sample; A_1_, the number of all haplotypes with one derived allele; A_2_, with two derived alleles, etc., such that ∑A_i_=n and not as *k_i_* opposite to ∑*Si*=*S*. Note that we have already introduced this notion to describe the allelic class *m=M* of the major haplotype (Table 1). In Figure 1, the theoretical distributions of the expected counts of haplotypes in each of their allelic classes m were computed using coalescent simulations under a simple population model. The plot of haplotype allelic classes in CDC25A summarizes well the characteristics of this segment revealed by the negative Tajima's and Fu Fs statistics as well as by Chakraborty's test. Due to the excess of haplotypes originating in polymorphisms with low counts of the derived alleles, the distribution of haplotype allelic classes is skewed to the left as compared to the simulated curve. CDC25A is representative of a group of loci sharing similar characteristics (Table 2), namely CDKN2A, MICA, SKP2, SMAD4, and, to a much lesser extent, HDAC1 (Supplementary Fig. S2).

On the other side of the spectrum are the segments with their major haplotypes removed by several mutations from the ancestral one, M≥3 (Table 1). As in CX3CR1 and GSTM3, in Figure 1 this time the observed distributions are skewed to the right relative to the simulated curve. As shown in Supplementary Figure S2, this group includes all segments already singled out by the Fay and Wu test and by the positive Fu and Li statistics (Table 2), i.e., BTN3A2, GPX3, GSTM3, GSTP1, and HTR2A, and in addition CX3CR1, which failed all these tests. Because recombinations were observed in some of these segments, we also simulated distributions of haplotype allelic classes assuming recombination at a rate 10 times the genomic average. It turned out that simulated distributions were relatively insensitive to crossovers, such that the observed distribution cannot be explained by the effect of recombinations at such intensity (dashed line in Fig. 1 for CX3CR1; see also Supplementary Fig. S2).

#### Population Variance;Fst

Over time, populations differentiate in allele frequencies, and the resulting geographic partitioning of this diversity can be measured by Fst [Wright, 1951].We estimated fixation index (Fst) values [Weir and Cockerham, 1984] for each segment based on the contributing sites and for each polymorphic site separately. Over the segments, the average Fst was 6.6±5.4% in the total sample composed of five population groups, and 4.3±4.5% when four non-African population groups were considered (Table 1). In turn, when 254 polymorphisms were considered individually, the average Fst was 4.6±5.8% for all populations and 3.1±5.1% considering only four non-African groups. The individual Fst values can be evaluated by comparison to their empirical distributions obtained from larger data sets [Akey et al., 2002; Excoffier, 2005; Fullerton et al., 2002], and this approach was used to single out promoter polymorphisms that presumably evolved under natural selection [Hahn et al., 2004; Rockman et al., 2003, 2004; Wang et al., 2005]. As a reference, we used our set ofthe 254 Fst values for polymorphisms investigated here (Supplementary Fig. S3). Several sites at the edge of the distribution exceeded the arbitrary threshold of the average Fst plus 2 SDs, either in the total five-population sample and/or in the non-African fourpopulation sample (Supplementary Table S4). Among them, we find three sites in CX3CR1—rs2669846:G>T, rs11715522:A>C, rs11917223:C>G—that are characterized by relatively elevated (30–50%) new allele frequencies. Interestingly, the same sites were not in the Hardy-Weinberg equilibrium (χ^2^=17.9, p<4×10^−5^; χ^2^=15.3, p<0.0001; χ^2^=8.3, p<0.005, in the world sample, and χ^2^=11.5, p<0.0009; χ^2^=10.6, p<0.005; χ^2^=9.6, p<0.005, in the non-African sample, respectively). Note that no other sites, with low or high Fst, showed any departure from the HWE, suggesting that this represents a specific effect of the CX3CR1 locus. Therefore, the Hardy-Weinberg disequilibrium together with high Fsts can be taken as evidence for evolutionary forces other than random drift acting upon this locus. On the other side, the Fst distribution, there are sites with zero or near zero Fsts. If the data are informative, this might suggest the effect of evolutionary forces countering random drift in order to maintain allele frequencies at similar levels across populations (e.g., BTN3A2; Table 1).

We note that the average per site Fst for all polymorphisms examined in this study was 4.6%, which is less than the corresponding values of 10 to 15% reported in the literature for a variety of genetic markers [Bowcock et al., 1991; Jorde et al., 2000; Akey et al., 2002]. This difference can be at least partly accounted for by the ascertainment bias and high average per site heterozygosity of the classical, early RFLP as well as Alu markers [Bowcock et al., 1991; Jorde et al., 2000]. Here, in contrast, allpolymorphic sites are considered, including those of low minor allele frequency [Ronald and Akey, 2005]. However, a similarly obtained (resequencing) set of 297 polymorphisms from the expressed sequence tags [Fan et al., 2002] shows twice as high an average Fst when compared with our data (9.2±12.7% for the total sample and 6.5±10.4% when Africans were excluded). Yet the shape of the Fst distribution is virtually identical in both data sets (Supplementary Fig. S3), raising the question whether the low average Fst observed here reflects its overall depression upstream of the protein-coding genes or only represents an artifact of a particular configuration of our population samples.

#### Haplotype Allelic Classes and the Prevalence of Major Haplotypes

**FIGURE 2:**
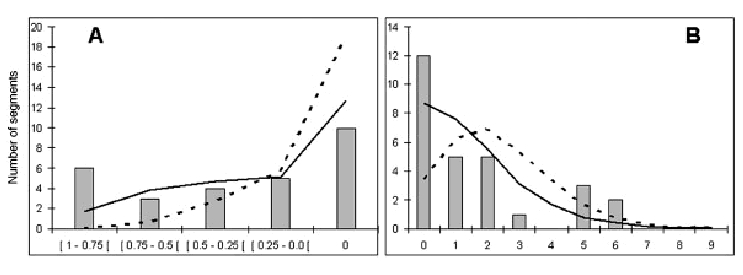
Frequencies of the ancestral haplotypes (**A**) and the distribution of major haplotypes among haplotype allelic classes (**B**) (cf. Fig.1) in 28 studied segments.The data (solid bars) are compared with theoretical expectations fromcoalescent simulations under the standard model in the absence (solid line) and in the presence (dashed line) of recombination at 10-fold the genomic average (10 cm/Mb). Simulations were for a sample size of 80 chromosomes, a mutation rate of 2.13×10^−8^ per bp per generation,corresponding to the average S density of 9.1, and *N* =10,000.

In our data, we found two opposite variation patterns at the level of haplotypes (see haplotype networks in Supplementary Fig. S1 and the list of haplotypes in Supplementary Table S3). The first pattern is characterized by the dominant presence of the ancestral haplotype (all of whose alleles are ancestral), whereas the second includes loci where the ancestral haplotype is absent or is present only at residual frequencies. These two opposite diversity profiles can be contrasted during analysis of all segments together. In Figure 2A, we show the histogram of the ancestral haplotype frequencies in our sample of 28 segments, counting loci with no ancestral haplotype and the number of those with the ancestral haplotype falling within each of the four frequency quartiles. The frequency “0” and the first quartile [0–0.25] represent 15 loci in a category of segments lacking or with ancestral haplotypes at minor frequencies. The three other quartiles [0.25–1.0] include 12 loci where the ancestral represents the major haplotype and CDKN1B segment and where two haplotypes, the ancestral one and the one carrying one derived allele, happened to have equal frequencies (M=0/1 in Table 1). Coalescent simulations were used to compare these data with theoretical distributions expected under a simple population model [Akey et al., 2004; Hudson, 1990; Schaffner et al., 2005]. We evaluated the expected distribution in 28 sequence segments, given the average density of polymorphic sites in this data set. Simulations were carried out under the constant population size in the absence and in the presence of recombinations (10-fold the genomic average). It turns out that in 28 segments analyzed we observe ancestral haplotypes more often than predicted under this model and that these ancestral haplotypes tend to occur at frequencies higher than expected. They prevail in the uppermost frequency quartile (Fig. 2A). The difference between the observed and the expected is even more acute in the presence of recombinations. On the other hand, to visibly affect the distribution, the recombinations would have to occur at a rate well above the genomic average.

In Figure 2B, the histogram of the partition of major haplotypes among haplotype allelic classes (i.e., *M* from Table 1) shows a U-like skewed distribution, as in Figure 2A. We observe an excess of the ancestral haplotypes (on the left) and of haplotypes carrying five or six new alleles, on the right of the distribution. The first effect becomes more pronounced in the presence of recombinations, while the second becomes attenuated but does not disappear in their presence. Our simulations also examined different demographic scenarios, such as those described by Akey et al. [2004], showing that neither population growth nor population bottlenecks substantially affected the simulated distributions with respect to the simple population model (Supplementary Fig. S4). In other words, among our loci there are more ancestral haplotypes that are major haplotypes than would be expected under the simple neutral model. At the same time, we observe a surplus of haplotypes being major and carrying excessive numbers of the derived alleles, consistent with the results of the statistical tests from Table 2. In summary, the analysis presented above suggests that in the investigated set of segments, certain loci appear as evolutionarily conserved while others seem to be more evolved relative to the average.

### DISCUSSION

We have analyzed 28 genomic segments located upstream from the transcription start sites of the protein-coding genes, where transcription control elements usually reside. The overall pattern of the observed diversity, both qualitatively and quantitatively, did not differ from the genomic average of nonexonic DNA segments. Nucleotide diversity of 0.082% was similar to that observed by others in noncoding sequences [Livingston et al., 2004; Zhao et al., 2000]. Likewise, the rate of evolution estimated from the sequence difference with chimpanzees (d=1.28%; µ=1.07×10^−9^ per bp per year) represented the genomic average [Chen and Li, 2001], leading to an estimate of the effective population size of 9,600, a value typically obtained in studies of human DNA diversity. However, large variances in different diversity indices, in summary statistics, and in distinct estimates of the population mutation parameter Θ (Table 1) suggested that the observed averages did not accurately reflect the extent of the investigated segments' diversities. This effect was particularly well captured by the analysis of the prevalence of the ancestral haplotypes and that of the major haplotypes among haplotype allelic classes (Fig. 2). In a large number of loci, the ancestral haplotype was a major haplotype, but at the same time, there were other, relatively numerous loci with M≥5 with rare or nonexistent ancestral haplotype. Together this led to a skewed, U-like distribution of the data plotted in Figure 2, showing departure from the neutral model based on its empirical evaluation by coalescence simulations. Therefore, it is tempting to propose that such a distribution is a product of the combined effects of the purifying selection acting on some of the loci and of adaptive evolution on the other loci. Indeed, significant results of statistical tests, such as that of Tajima (negative D) or Fay and Wu (negative H), are consistent with these opposite selection effects. On the other hand, the incongruence in different diversity estimators revealed by neutrality tests can be also ascribed to the demographic history itself affecting gene genealogies even in the absence of selection. It is usually argued that selection acts upon specific loci, while demography is common to all genomic segments and thus should affect them in the same way Akey et al., 2004; Reed et al., 2005]. As a result, for loci sampled from the same population, the associated variance, due to shared demography, is expected to be lower and the detection of selection easier. Nevertheless, one has to consider that natural selection likely acts on different loci at different time periods and that the resulting diversity patterns are also differently and randomly affected by their genealogical histories. Given all this, when postulating the effect of selection, the results of different tests and different descriptive statistics, as well as geographic distribution of the genetic variants, should be considered together, including data on functional testing, if available.

A set of loci plausibly contributing to the observed excess of ancestral haplotypes among major haplotypes include CDC25A, CDKN2A, and SMAD4. These segments remained significant for negative Tajima's *D* (and negative D* of Fu and Li in the case of SMAD4) after correction for multiple testing. The interpretation of the haplotype-diversity-based tests, Fu's Fs and Chakraborty's statistics (Table 2) is more complicated. While corroborating Tajima's test in the case of the segments above, in the case of GPX2 the significant results can also be ascribed to the effect of recombinations and/or to the population amalgamation [Chakraborty, 1990]. The population amalgamation can be also invoked in the equally diverse MICA and HDAC1, where the effect of recombination can be neglected. In other words, a plausible genetic (recombination) or demographic (population structure) explanation for the observed diversity patterns can be proposed in these loci even in the absence of selection and despite that these data originate from the same population sample as those from other loci analyzed here. On the other side of the diversity spectra (Table 1; Figs. 1 and 2) are the segments with elevated YH and M. They include BTN3A2, GPX3, GSTM3, and HTR2A, which were initially singled out by the Fay and Wu test; GSTP1, which remained significant for Fu and Li statistics after correction (Table 2); and CX3CR1. The latter was singled out by the departure from HWE and Fst values of its three segregating sites as well as by its distribution in the plot of haplotype allelic classes. Moreover, with S=16, Θ_π_=4.03 (i.e., π=0.20%, 2 SD above the average), k=17, G=0.86, and Θ_k_=6.032, CX3CR1was the most diversified segment among those analyzed in this study and the only one that turned out in the HKA test (p<0.025; in which we compared each of the segments against the collection of 28 segments analyzed here). In these six loci, we observed an important skew toward high haplotype allelic classes (Fig. 1; Supplementary Fig. S2) compared to the neutral expectation under a simple population model. Consequently, all of them but GSTM3 (M=3) also contributed to a rightward skew of the data plot in Figure 2 that could not be explained by the demographic scenarios proposed by Akey et al. [2004] and only to some extent by recombination, although only at well above the average genomic rate.

The question is, to what extent are our findings concerning 5′ flanking regions particular to these segments and to what extent are they representative for other noncoding 2-kb sequences. One can also argue that our sample is biased due to a particular set of genes we examined and therefore not representative for the other 5′ flanking sequences. But this is almost admitting that these loci are special, indirectly reinforcing a selectionist interpretation, whereby the effects of purifying and adaptive selection did interchangeably create opposite patterns of diversity. In any case, our results provide a useful reference for future comparative analyses that will eventually show to what extent the observed variance in genetic diversity among sequence segments reflects the genomic reality and what part is attributable to selection, to stochastic effects, and to complex demographic histories. On the other hand, additional data will be required to dissociate the effects particular to the examined region from the influence of the linked, adjacent sequences. Indeed, there are numerous reports describing promoter regions as containing variant sites affecting their function and as containing variants associated with a disease or representing likely targets of selection. In this context, it is interesting to note that polymorphic site rs769214 in the CAT promoter (see Supplementary Table S2) was reported to be associated with different blood pressure levels (originally SNP844 in Jiang et al. [2001]). In turn, the new allele of the rs36228834 site in CDKN2A and the ancestral allele of the rs36228499 polymorphisms in CDKN1B were found to be associated with an increased risk of childhood acute lymphoblastic leukemia [Healy et al., 2006]. At another site in CDKN1B, the new T allele of rs3759217, with relatively high Fst in non-African populations, abolishes the myoblast-determining-factor binding site [Lassar et al., 1989], i.e., CANCtg→TANCtg (see TRANSFAC; www.gene-regulation.com/pub/databases.html), although the relevance of this mutation will have to be confirmed experimentally. In BTN3A2, with one dominant (68%) haplotype (Supplementary Figs. S1 and S2) carrying five derived alleles and conspicuously zero Fst (Table 1), a selection sweep preceding expansion of human populations could be suggested. Here, an adaptive change could have been associated with an increased transcription rate. By allelic-imbalance experiments [Pastinen et al., 2004], the expression of the ancestral haplotype was previously shown to be relatively suppressed. The same effect was demonstrated independently by in vitro transcription (N'Diaye, Pastinen, Paterson, Larivie`re, Labuda, Hudson, and Sinnett, unpublished data) of cloned constructs of the 2-kb BTN3A2 haplotypes from the present study. Similarly, in GSTM3, the functional analysis of its rs1332018G>T polymorphism (originally −63 A/C in Liu et al. [2005]) has shown eight-fold lower transcription activity of the G allele related to its nine-fold reduced RNA Pol II binding capacity [Liu et al., 2005]. This ancestral G is absent in Africans but occurs at high frequencies outside Africa. In turn, in HTR2A and TGFB1, strong purifying selection on their coding segments was reported by Bustamante et al. [2005], and it appears not to be reflected in diversity profiles of their 5′ flanking sequence (Tables 1 and 2). In contrast, in IL1A, the effect of local adaptation postulated from the analysis of the whole gene by Akey et al. [2004], seems to be reflected by the presence of three equally prevalent haplotypes of this segment, consistent with the scenario of balancing selection. In fact, a newer version of its mRNA (REFSEQ NM_000575.3) shifts the transcription start sites by 1.6 kb and all its polymorphisms are now found within the 5′ portion of the gene and none within the 0.4-kb upstream sequence analyzed here. We note also a possible shift in the promoter region of the CX3CR1 locus (NM_001337.3), with its new first exon located about 13 kb upstream with respect to the NM_001337.1 mRNA version we used. In contrast, the transcription start sites of the remaining genes either stayed the same or changed the position of the analyzed 2-kb segment by less than 0.2 kb.

In their recent work, Di Rienzo and Hudson [2005] proposed an evolutionary framework for common diseases, listing numerous examples where the ancestral allele represented a susceptibility variant. As shown above, such susceptibility alleles could be found in the promoter regions (CDKN1B) or represent plausible susceptibility candidates, conferring different expression activities and/or differing in geographic occurrence (e.g., BTN3A2 and GSTM3). If adaptive evolution is preferably regulatory [King and Wilson, 1975] and still prevalent in human populations, more regulatory variants could be expected in the promoter as opposed to in the coding segments. Our study provides new evidence suggesting the role of selection, including adaptive changes in the evolution of these segments. However, because molecular signatures of selection are relatively weak and our segments are short, selectionist interpretations have to be considered with caution. Nevertheless, recent findings point to the evolutionary importance of the cis-acting regulatory elements [Carroll, 2005; Rockman et al., 2005], and our results add to the increasing evidence suggesting that positive selection may be more pervasive in the human population than previously thought [Nielsen, 2005].
