# Supplementary material for: Patterns of variation in DNA segments upstream of transcription start sites
Source: Hum Mutat. 2007 May;28(5):441–50. doi: 10.1002/humu.20463 (PMC2683062; doi:10.1002/humu.20463)
Supplement: Supplementary file 1 [file humu0028-0441-SD1.doc]

***Supplementary Material for the article:***

Labuda et al., ***Human Mutation***

**Patterns of Variation in DNA Segments Upstream of the Transcription Start Sites**

Damian Labuda, Catherine Labbé, Sylvie Langlois, Jean-Francois Lefebvre, Virginie Freytag,
Claudia Moreau, Jakub Sawicki, Patrick Beaulieu, Tomi Pastinen, Thomas J. Hudson,
and Daniel Sinnett

### Supplementary Tables S1-S4

### Supplementary Figures S1-S4

**Supplementary Table S1:** **Investigated Loci**

| Gene Symbol | Gene Name | Cytogenetic Position | mRNA REFSEQ |
| --- | --- | --- | --- |
| BTN3A2 | butyrophilin, subfamily 3, member A2 | 6p22.1 | NM_007047.1 |
| CAT | catalase | 11p13 | NM_001752.1 |
| CCND1 | cyclin D1 (PRAD1: parathyroid adenomatosis 1) | 11q13 | NM_053056.1 |
| CCNE1 | cyclin E1 | 19q12 | NM_001238.2 |
| CDC25A | cell division cycle 25A | 3p21 | NM_001789.1 |
| CDKN1A | cyclin-dependent kinase inhibitor 1A (p21, Cip1) | 6p21.2 | NM_000389.2 |
| CDKN1B | cyclin-dependent kinase inhibitor 1B (p27, Kip1) | 12p13.1-p12 | NM_004064.2 |
| CDKN2A | cyclin-dependent kinase inhibitor 2A (melanoma, p16, inhibits CDK4) | 9p21 | NM_000077.2 |
| CX3CR1 | chemokine (C-X3-C) receptor 1 | 3p21.3 | NM_001337.1 |
| E2F1 | E2F transcription factor 1 | 20q11.2 | NM_005225.1 |
| FEN1 | flap structure-specific endonuclease 1 | 11q12 | NM_004111.3 |
| FGB | fibrinogen, B beta polypeptide | 4q28 | NM_005141.1 |
| GPX2 | glutathione peroxidase 2 | 14q24.1 | NM_002083.1 |
| GPX3 | glutathione peroxidase 3 | 5q23 | NM_002084.2 |
| GSS | glutathione synthetase | 20q11.2 | NM_000178.1 |
| GSTM3 | glutathione S-transferase M3 | 1p13.3 | NM_000849.1 |
| GSTM4 | glutathione S-transferase M4 | 1p13.3 | NM_000850.1 |
| GSTP1 | glutathione S-transferase pi | 11q13-qter | NM_000852.2 |
| HDAC1 | histone deacetylase 1 | 1p34 | NM_004964.2 |
| HTR2A | 5-hydroxytryptamine (serotonin) receptor 2A | 13q14-q21 | NM_000621.1 |
| IL1A | interleukin 1, alpha | 2q14 | NM_000575.1 |
| MICA | MHC class I polypeptide-related sequence A | 6p21.3 | NM_000247.1 |
| RB1 | retinoblastoma 1 (including osteosarcoma) | 13q14.2 | NM_000321.1 |
| SKP2 | S-phase kinase-associated protein 2 (p45) | 5p13 | NM_005983.2 |
| SMAD3 | MAD, mothers against decapentaplegic homolog 3 | 15q21-q22 | NM_005902.1 |
| SMAD4 | MAD, mothers against decapentaplegic homolog 4 | 18q21.1 | NM_005359.2 |
| TFDP1 | transcription factor Dp-1 | 13q34 | NM_007111.2 |
| TGFB1 | transforming growth factor, beta 1 | 19q13.2 | NM_000660.1 |

**Supplementary Table S2: List of segregating sites and the corresponding allele-specific**

**oligonucleotide** ASO probes

| Segment name | Position | Rs indentifier | Total sequence |
| --- | --- | --- | --- |
| **BTN3A2** | -113 | rs3734536 | TGAGGAA**C**GTTGAAA |
|  |  |  | TGAGGAA**G**GTTGAAA |
|  | -133 | rs36222428 | ACTGAAA**G**TACATTG |
|  |  |  | ACTGAAA**C**TACATTG |
|  | -283 | rs36222429 | ATGGTAA**T**AGAAATA |
|  |  |  | ATGGTAA**C**AGAAATA |
|  | -312 | rs9393709 | TAGGAAA**G**TAAAAAAA |
|  |  |  | TAGGAAA**A**TAAAAAAA |
|  | -321 | rs36222431 | AAAAAAA**T**TTAATGTG |
|  |  |  | AAAAAAA**A**TTAATGTG |
|  | -479 | rs36222433 | TTAAGAA**C**TTCTGGC |
|  |  |  | TTAAGAA**A**TTCTGG- |
|  | -529 | rs9379855 | CTGGAAA**T**CAGAAAA |
|  |  |  | CTGGAAA**C**CAGAAAA |
|  | -812 | rs36222766 | CAGCCCA**A**AAAAGTG |
|  |  |  | CAGCCCA**G**AAAAGTG |
|  | -939 | rs12194095 | GCATGCA**C**GCTGCAGA |
|  |  |  | GCATGCA**T**GCTGCAGA |
|  | -1002 | rs36222767 | TCAAGGG**G**GAAGAGC |
|  |  |  | TCAAGGG**T**GAAGAGC |
|  | -1403 | rs10946817 | TGAAAAA**C**AAACTT |
|  |  |  | TGAAAAA**T**AAACTT |
|  | -1494 | rs36222768 | GAAGGGGGA**G**GGAAA- |
|  |  |  | GAAGGGGGA**A**GGGAAA |
|  | -1733 | rs36222771 | TGAGATC**G**TATTAGT |
|  |  |  | TGAGATC**A**TATTAGT |
|  | -1771 | rs9358933 | CTGGAGG**G**TCCAAGT |
|  |  |  | CTGGAGG**A**TCCAAGT |
| **CAT** | -21 | rs7943316 | CCCCGAG**T**CCGTGGC |
|  |  |  | CCCCGAG**A**CCGTGGC |
|  | -262 | rs17879971 | TGCCCGG**G**ATAGCCG |
|  |  |  | TGCCCGG**A**ATAGCCG |
|  | -465 | rs17883920 | AGCGTAT**G**GCAAAAT |
|  |  |  | AGCGTAT**A**GCAAAAT |
|  | -776 | rs769214 | CCTGGG**G**GTAAAATTTG |
|  |  |  | CCTGGG**A**GTAAAATTTG |
|  | -975 | rs9282626 | GATGGTA**T**ATACTATG |
|  |  |  | GATGGTA**C**ATACTATG |
|  | -1099 | rs36224102 | GTATGATA**A**GAATCTTG |
|  |  |  | GTATGATA**G**GAATCTTG |
|  | -1632 | rs564250 | TAATGAA**T**AAAGGTT |
|  |  |  | TAATGAA**C**AAAGGTT |
| **CCND1** | -519 | rs36225064 | TTAGTT**C**TTGCAATT |
|  |  |  | TTAGTT**T**TTGCAATT |
|  | -568 | rs36064048 | AAACTTGC**A**CAGGGGTT |
|  |  |  | AAACTTGC**C**CAGGGGTT |
|  | -766 | rs3212860 | AGGAAGGC**A**GCCCGAAG |
|  |  |  | AGGAAGGC**T**GCCCGAAG |
|  | -1537 | rs36225395 | GTCCTGGGGGG**GT**A**A** |
|  |  |  | GTCCTGGGGGG**TA**A**T** |
|  | -1554 | rs36225394 | GGAATCC**G**GGGGTAA |
|  |  |  | GGAATCC**A**GGGGTAA |
|  | -1597 | rs36225073 | AGGGAGCA**C**CTACGGTC |
|  |  |  | AGGGAGCA**T**CTACGGTC |
|  | -1701 | rs36225070 | GAATGAAAAA**G**CAG |
|  |  |  | GAATGAAAAA**C**CAG |
|  | -1737 | rs36225069 | CATCC**A**GGCGGCCGC |
|  |  |  | CATCC**T**GGCGGCCGC |
|  | -1885 | rs36225068 | CGGAGT**G**GGCGAGC |
|  |  |  | CGGAGT**T**GGCGAGC |
|  | -1888 | rs36225067 | GCCGGCGT**G**GGCG |
|  |  |  | GCCGGCGT**T**GGCG |
|  | -1926 | rs36225072 | GATGGGGGGG**-**TGAAGCT |
|  |  |  | GATGGGGGGG**G**TGAAGCT |
|  | -1931 | rs36225071 | GGATG**C**GGGGGTGAAG-- |
|  |  |  | GATG**G**GGGGGTGAAGCT |
|  | -1938 | rs1944129 | TTGCGCC**C**GGGATG |
|  |  |  | TTGCGCC**T**GGGATG |
| **CCNE1** | -1366 | rs36225398 | ATAGTGC**C**TGGACCA |
|  |  |  | ATAGTGC**T**TGGACCA |
|  | -1613 | rs36225399 | GCTCTAACT**T**CAAGAAA |
|  |  |  | GCTCTAACT**-**CAAGAAA |
| **CDC25A** | -51 | rs3731485 | CTTTCCTA**G**TTGGCGC |
|  |  |  | CTTTCCTA**C**TTGGCGC |
|  | -171 | rs3731484 | AGAGCCGA**T**GACCTGGC |
|  |  |  | AGAGCCGA**A**GACCTGGC |
|  | -351 | rs3731482 | CCAGTTA**G**CTTCGGC |
|  |  |  | CCAGTTA**A**CTTCGGC |
|  | -1433 | rs6809142 | GCTCAACAGGC**C**GGG |
|  |  |  | GCTCAACAGGC**T**GGG |
|  | -1440 | rs34440842 | GGGAGC**G**GTGGCTC |
|  |  |  | GGGAGC**A**GTGGCTC |
|  | -1566 | rs6763242 | TTAC**C**GGGCGTGGTGG |
|  |  |  | TTAC**T**GGGCGTGGTGG |
|  | -2030 | rs1903061 | TGGGGCTGG**T**GGGAAGT |
|  |  |  | TGGGGCTGG**G**GGGAAGT |
| **CDKN1A** | -134 | rs4135239 | -CTGGCTC**G**GCGCTGG |
|  |  |  | GCTGGCTC**C**GCGCTGG |
|  | -499 | rs4151702 | GGGAATA**G**AGGTGAT |
|  |  |  | GGGAATA**C**AGGTGAT |
|  | -519 | rs730506 | TGACCTA**G**TGAGGGA |
|  |  |  | TGACCTA**C**TGAGGGA |
|  | -791 | rs2395655 | GAAGAAA**T**CCCTGTG |
|  |  |  | GAAGAAA**C**CCCTGTG |
|  | -899 | rs762624 | GGGGAAAC**T**GGGGCTCA |
|  |  |  | GGGGAAAC**G**GGGGCTCA |
|  | -1021 | rs762623 | ATTTTGGA**G**CCACAGAA |
|  |  |  | ATTTTGGA**A**CCACAGAA |
|  | -1284 | rs733590 | ATCCCACT**A**AAAAACAG |
|  |  |  | ATCCCACT**G**AAAAACAG |
|  | -1466 | rs3829968 | GACACAG**T**AGCAGAC |
|  |  |  | GACACAG**C**AGCAGAC |
|  | -1529 | rs3829967 | GTGGCC**T**TTCTGG |
|  |  |  | GTGGCC**C**TTCTGG |
|  | -1536 | rs3829966 | GGCCAC**G**GCACAGC |
|  |  |  | GGCCAC**A**GCACAGC |
|  | -1731 | rs36228198 | GGAGAAA**A**TTTTTTTTA |
|  |  |  | GGAGAAA**-**TTTTTTTTA |
| **CDKN1B** | -221 | rs36228495 | AGAGCAGG**T**TTGTTGGC |
|  |  |  | AGAGCAGG**C**TTGTTGGC |
|  | -337 | rs36228498 | GGGTTCGC**C**GCGGCG |
|  |  |  | GGGTTCGC**A**GCGGCG |
|  | -373 | rs36228499 | TTCCCGGG**C**GAGGAGC |
|  |  |  | TTCCCGGG**A**GAGGAGC |
|  | -522 | rs248743 | CTGGGGG**G**GCTGGGC |
|  |  |  | CTGGGGG**A**GCTGGGC |
|  | -756 | rs36228497 | TTAAAAAA**A**AAAAAC |
|  |  |  | TTAAAAAA**C**AAAAAC |
|  | -1084 | rs36228201 | AGAGGGCTG**AAGGGTG** |
|  |  |  | AGAGGGCTG**GGTGAAA** |
|  | -1228 | rs36228202 | AGTGTGG**G**CTTGAGC |
|  |  |  | AGTGTGG**A**CTTGAGC |
|  | -1608 | rs35756741 | TGTCCAGG**G**ACATGCAT |
|  |  |  | TGTCCAGG**A**ACATGCAT |
|  | -1857 | rs3759217 | CTCAAGA**C**AGCTGCA |
|  |  |  | CTCAAGA**T**AGCTGCA |
|  | -1908 | rs36228203 | GAAGGAC**A**GGG**G**T**TG** |
|  |  |  | GAAGGAC**G**GGG**T**T**GA** |
| **CDKN2A** | -44 | rs36228836 | CAGAGCC**A**GCGTTGGCA |
|  |  |  | CAGAGCC**T**GCGTTGGCA |
|  | -222 | rs36228834 | GGCAGTT**A**GGAAGGT |
|  |  |  | GGCAGTT**T**GGAAGGT |
|  | -464 | rs3731238 | AGGGGAAG**G**AGAGAGCA |
|  |  |  | AGGGGAAG**A**AGAGAGCA |
|  | -589 | rs36228501 | GACGGGG**G**AGAATTC |
|  |  |  | GGACGGGG-AGAATTC |
|  | -598 | rs36228502 | GAGAATTCT**G**CCTGTAG |
|  |  |  | GAGAATTCT**A**CCTGTAG |
|  | -710 | rs36228503 | AGAGTTG**C**ACAGTGA |
|  |  |  | AGAGTTG**A**ACAGTGA |
|  | -1600 | rs36228838 | CTGGGAG**A**CAAGAGC |
|  |  |  | CTGGGAG**G**CAAGAGC |
|  | -1879 | rs3731236 | GGATTATC**C**AGTAGGCA |
|  |  |  | GGATTATC**T**AGTAGGCA |
| **CX3CR1** | -36 | rs230801 | TACCAGA**G**ACGAGTA |
|  |  |  | TACCAGA**A**ACGAGTA |
|  | -58 | rs36230802 | CTTAGAG**G**GAAGGAA |
|  |  |  | CTTAGAG**T**GAAGGAA |
|  | -302 | rs36230798 | GCAGTCA**G**CAAAGCA |
|  |  |  | GCAGTCA**A**CAAAGCA |
|  | -325 | rs36230797 | CCATTAATG**A**GAGCTG |
|  |  |  | CCATTAATG**G**GAGCTG |
|  | -387 | rs871610 | CTGCCCA**C**GAGAGGG |
|  |  |  | CTGCCCA**T**GAGAGGG |
|  | -738 | rs36230800 | AGAGACT**G**TGAAAGG |
|  |  |  | AGAGACT**A**TGAAAGG |
|  | -986 | rs871144 | GGGTCAC**A**GACCATT |
|  |  |  | GGGTCAC**G**GACCATT |
|  | -1185 | rs938203 | TCCACCA**T**GAGCAGG |
|  |  |  | TCCACCA**C**GAGCAGG |
|  | -1242 | rs9818167 | AAAGGTG**G**CCAAACA |
|  |  |  | AAAGGTG**A**CCAAACA |
|  | -1346 G/T | rs2669846 | GTATCA**AA**GGGTGGT |
|  |  |  | GTATCA**CG**GGGTGGT |
|  | -1345 C/T | rs2853709 | GTATCA**AG**GGGTGGT |
|  |  |  | GTATCA**CA**GGGTGGT |
|  | -1363 | rs2853708 | GAGTGGA**C**CCCAGGA |
|  |  |  | GAGTGGA**G**CCCAGGA |
|  | -1469 | rs36230461 | TGTGGGA**C**AGGAAGA |
|  |  |  | TGTGGGA**A**AGGAAGA |
|  | -1484 | rs36230460 | GACTGTC**G**TCAAGAG |
|  |  |  | GACTGTC**C**TCAAGAG |
|  |  |  | GACTGTC**A**TCAAGAG |
|  | -1683 | rs11715522 | CCAACTT**A**AACGCCT |
|  |  |  | CCAACTT**C**AACGCCT |
|  | -1943 | rs11917223 | GACACTG**G**ACGCTGG |
|  |  |  | GACACTG**C**ACGCTGG |
| **E2F1** | -187a | rs3213141 | CGCGCC**C**CGCCCCG |
|  |  |  | CGCGCC**T**CGCCCCG |
|  | -243 | rs36231779 | CCGGGC**T**GGGGGC |
|  |  |  | CCGGGC**G**GGGGGC |
|  | -300 | rs36231778 | GCGTCGG**G**GCGGGG |
|  |  |  | GCGTCGG**A**GCGGGG |
|  | -897 | rs3213134 | GAGATGG**G**TCTTCAC |
|  |  |  | GAGATGG**A**TCTTCAC |
| **FEN1** | -69 | rs174538 | CACGTCG**G**CAGGAGC |
|  |  |  | CACGTCG**A**CAGGAGC |
|  | -704 | rs695865 | GGAAGG**G**GGCCGTGA |
|  |  |  | GGAAGG**A**GGCCGTGA |
|  | -1688 | rs36203060 | CCCGGGC**G**ACAGAGT |
|  |  |  | CCCGGGC**A**ACAGAGT |
|  | -1733 | rs36203061 | GTCAAAAAAA**AAA**GATATTG |
|  |  |  | GTCAAAAAAA**---**GATATTG |
|  | -1804 | rs36203059 | GGACCAG**T**GTCTATA |
|  |  |  | GGACCAG**C**GTCTATA |
| **FGB** | -148 | rs1800787 | GTAAATAAG**C**TTTGCTG |
|  |  |  | GTAAATAAG**T**TTTGCTG |
|  | -199 | rs36203396 | GTTTAGAAA**C**ATTAAA |
|  |  |  | GTTTAGAAA**T**ATTAAA |
|  | -249 | rs1800788 | AGGAGTG**C**CCTAACT |
|  |  |  | AGGAGTG**T**CCTAACT |
|  | -455 | rs1800790 | GGGGC**C**ATTAAAATC |
|  |  |  | GGGGC**T**ATTAAAATC |
|  | -727 | rs36203400 | CAATAA**A**ATAGAATG |
|  |  |  | CAATAA**T**ATAGAATG |
|  | -854 C/G | rs1800791 | GTGG**G**AAAT**C**AAGGA |
|  |  |  | GTGG**A**AAAT**G**AAGGA |
|  | -849 G/A | rs36203399 | GTGG**A**AAAT**C**AAGGA |
|  |  |  | GTGG**G**AAAT**G**AAGGA |
|  | -993 | rs2227389 | CATTTTTCT**G**TTTTTTG |
|  |  |  | CATTTTTCT**A**TTTTTTG |
|  | -1143 | rs2227388 | CTGGAGA**A**GAAAGAA |
|  |  |  | CTGGAGA**G**GAAAGAA |
|  | -1420 | rs1800789 | GCAAATAT**C**TATGTGGC |
|  |  |  | GCAAATAT**T**TATGTGGC |
|  | -1552 | rs36203403 | TAACAGATA**A**TTCTTT |
|  |  |  | TAACAGATA**G**TTCTTT |
|  | -1832 | rs36203766 | GAATGTTAA**G**TCTGTG |
|  |  |  | GAATGTTAA**A**TCTGTG |
|  | -1852 | rs2227385 | CAAGAACT**A**TTTGTAAG |
|  |  |  | CAAGAACT**G**TTTGTAAG |
| **GPX2** | -166 | rs17883620 | TCAGTCTG**T**TAATAGGA |
|  |  |  | TCAGTCTG**C**TAATAGGA |
|  | -199 | rs36206353 | CACC**C**TGCTGCAACC |
|  |  |  | CACC**G**TGCTGCAACC |
|  | -360 | rs36206674 | CGTCTGTG**T**CAAATGAG |
|  |  |  | CGTCTGTG**C**CAAATGAG |
|  | -442 | rs36206677 | TCTGAAAG**A**TGAAGAAG |
|  |  |  | TCTGAAAG**G**TGAAGAAG |
|  | -512 | rs17093568 | GAAGAAC**G**TGAACAG |
|  |  |  | GAAGAAC**T**TGAACAG |
|  | -524 | rs17880939 | AAAGCAAC**A**AAGAGAAG |
|  |  |  | AAAGCAAC**G**AAGAGAAG |
|  | -724 | rs3831077 | AATATAGG-GATGGTACA |
|  |  |  | AATATAGG**A**GATGGTAC- |
|  | -843 | rs17884169 | GTGGATGA**A**CTGCACCT |
|  |  |  | GTGGATGA**G**CTGCACCT |
|  | -1070 | rs2296327 | CAGAAAC**C**GAAAAGA |
|  |  |  | CAGAAAC**T**GAAAAGA |
|  | -1195 | rs36206681 | TTTCAATAA**C**AAAGC |
|  |  |  | TTTCAATAA**T**AAAGC |
|  | -1276 | rs17881260 | AGCAAGTC**C**GGGGAGCA |
|  |  |  | AGCAAGTC**T**GGGGAGCA |
|  | -1659 | rs36206974 | CAAAAGAGC**A**GAGAATA |
|  |  |  | CAAAAGAGC**G**GAGAATA |
|  | -1723 | rs4902347 | GGAAGAGG**G**AACCCCCA |
|  |  |  | GGAAGAGG**A**AACCCCCA |
|  | -1913 | rs36206977 | TAAGCAGC**G**TATGATTC |
|  |  |  | TAAGCAGC**A**TATGATTC |
|  | -2022 | rs3825644 | GAGAATTG**T**TTGTAGGA |
|  |  |  | GAGAATTG**G**TTGTAGGA |
|  | -2077 | rs36206975 | CAC**T**GAATTGGAATC |
|  |  |  | CAC**C**GAATTGGAATC |
| **GPX3** | -51 | rs6888961 | CAGGGCA**A**GGGGGCTAA |
|  |  |  | CAGGGCA**T**GGGGGCTAA |
|  | -69 | rs8177409 | TCAGTTCT**A**GGGAGCTA |
|  |  |  | TCAGTTCT**T**GGGAGCTA |
|  | -285 | rs8177406 | GGGCATT**A**GGACTGT |
|  |  |  | GGGCATT**G**GGACTGT |
|  | -335 | rs8177404 | CATAAGG**A**TCACAGC |
|  |  |  | CATAAGG**G**TCACAGC |
|  | -617 | rs8177402 | TGGCTTA**G**AAGCAGA |
|  |  |  | TGGCTTA**A**AAGCAGA |
|  | -628 | rs1946236 | TTT**A**TTTTCTGCTTCT |
|  |  |  | TTT**T**TTTTCTGCTTCT |
|  | -694 | rs1946235 | CATTACA**T**AGCTGAGG |
|  |  |  | CATTACA**C**AGCTGAGG |
|  | -709 | rs1946234 | GATGGGAT**T**GATTAGTG |
|  |  |  | GATGGGAT**G**GATTAGTG |
|  | -776 | rs36207266 | GACAGAC**G**GAGACCA |
|  |  |  | GACAGAC**A**GAGACCA |
|  | -1958 | rs3763013 | TTGCTAC**G**GAAAGTA |
|  |  |  | TTGCTAC**A**GAAAGTA |
|  | -2005 | rs2042235 | GTCCACCT**A**TTTAAC |
|  |  |  | GTCCACCT**G**TTTAAC |
| **GSS** | -514 | rs3761144 | CTGGGATA**C**CAGCAGGG |
|  |  |  | CTGGGATA**G**CAGCAGGG |
|  | -716 | rs36208867 | AGGTGCCTTCC**C**TGTTT |
|  |  |  | AGGTGCCTTCC**G**TGTTT |
|  | -792 | rs36208520 | ATGGAAT**G**TATCAGT- |
|  |  |  | ATGGAAT-TATCAGTC |
|  | -1125 | rs36208521 | TAGAGAC**G**GGGTTTC |
|  |  |  | TAGAGAC**A**GGGTTTC |
|  | -1412 | rs36208522 | CAACAGG**A**CTTAGGC |
|  |  |  | CAACAGG**T**CTTAGGC |
|  | -1494 | rs13041792 | AGGGGAA**G**GACATGG |
|  |  |  | AGGGGAA**A**GACATGG |
|  | -1762 | rs36208864 | AACAACAA**C**GAAAACTC |
|  |  |  | AACAACAA**T**GAAAACTC |
|  | -1943 | rs7268769 | CAGACCAA**T**CTGGCCAA |
|  |  |  | CAGACCAA**C**CTGGCCAA |
|  | -1979 | rs3787212 | TCCACCC**G**CCCCAGC |
|  |  |  | TCCACCC**A**CCCCAGC |
| **GSTM3** | 17 G/A | rs36210413 | GACAT**A**GT**G**ACGGGCTT |
|  |  |  | GACAT**A**GT**A**ACGGGCTT |
|  | 14 A/G | rs36210754 | GACAT**G**GT**G**ACGGGCTT |
|  |  |  | GACAT**G**GT**A**ACGGGCTT |
|  | -46 | rs1332018 | TATACCC**G**ACATAAG |
|  |  |  | TATACCC**T**ACATAAG |
|  | -208 | rs36210756 | AGGAGTG**C**TGCAGGA |
|  |  |  | AGGAGTG**G**TGCAGGA |
|  | -859 | rs4970777 | TAAATGC**G**TGAGCAT |
|  |  |  | TAAATGC**A**TGAGCAT |
|  | -1286 | rs4970737 | CACAGCA**G**AGTGGCT |
|  |  |  | CACAGCA**C**AGTGGCT |
| **GSTM4** | -22 | rs1010167 | TCCGGAA**G**CACTAGG |
|  |  |  | TCCGGAA**C**CACTAGG |
|  | -195 | rs36210760 | AGCCGAGA**G**GCAGAAGG |
|  |  |  | AGCCGAGA**C**GCAGAAGG |
|  | -584 | rs36210763 | TAAGAA**T**GTGTCATT |
|  |  |  | TAAGAA**C**GTGTCATT |
|  | -821 T/C | rs36211084 | ACTGGT**CTT**GAACTC |
|  |  |  | ACTGGT**CTC**GAACTC |
|  | -822 T/G | rs36211085 | ACTGGT**CGC**GAACTC |
|  |  |  | ACTGGT**CGT**GAACTC |
|  | -823 C/T | rs6686777 | ACTGGT**TT**TGAACTC |
|  |  |  | ACTGGT**TG**TGAACTC |
|  |  |  | ACTGGT**TT**CGAACTC |
|  |  |  | ACTGGT**TG**CGAACTC |
|  | -1196 | rs36210761 | AAAATA**C**GGGACACT |
|  |  |  | AAAATA**G**GGGACACT |
|  | -1302 | rs641592 | TGAGGAGC**C**AGGAAGAC |
|  |  |  | TGAGGAGC**T**AGGAAGAC |
|  | -1614 G/A | rs542370 | GCCTTTCC**A**TA**G**TTG |
|  |  |  | GCCTTTCC**G**TA**G**TTG |
|  | -1617 A/G | rs3754450 | GCCTTTCC**A**TA**A**TTG |
|  |  |  | GCCTTTCC**G**TA**A**TTG |
| **GSTP1** | -189 | rs8191438 | CGGCCAG**C**TGCGCGG |
|  |  |  | CGGCCAG**G**TGCGCGG |
|  | -280 | rs36211089 | AGTGTGC**G**CAGCGAA |
|  |  |  | AGTGTGC**A**CAGCGAA |
|  | -285 | rs36211088 | GCCAGTT**C**GCTGCGC |
|  |  |  | GCCAGTT**A**GCTGCGC |
|  | -286 | rs11311625 | CAGT**T**CGCTGCGCAC**-** |
|  |  |  | CAGT-CGCTGCGCACA |
|  | -564 | rs8191437 | AGCCGCC**G**AGATCGC |
|  |  |  | AGCCGCC**C**AGATCGC |
|  | -828 | rs4147580 | GTACATGT**A**GAGGATGT |
|  |  |  | GTACATGT**G**GAGGATGT |
|  | -829 | rs7109914 | ACATGTA**G**AGGATGT |
|  |  |  | ACATGTA**C**AGGATGT |
|  | -1231 | rs8191431 | AAGCCAA**C**AAGAGAT |
|  |  |  | AAGCCAA**T**AAGAGAT |
|  | -1386 | rs6591256 | GTTTTAA**A**GATACAA |
|  |  |  | GTTTTAA**G**GATACAA |
|  | -1736 | rs6591255 | CCAAAAC**A**GAAATAA |
|  |  |  | CCAAAAC**T**GAAATAA |
|  | -1937 | rs7949587 | AACCTGC**G**GACATGT |
|  |  |  | AACCTGC**A**GACATGT |
|  | -1964 | rs7949394 | GGTTTTA**C**GGCTCAG |
|  |  |  | GGTTTTA**T**GGCTCAG |
|  | -2088 | rs36211399 | TAGTCTAGTCTAGTT |
|  |  |  | ATTCTAGTTTCGCCG |
| **HDAC1** | -162 | rs36212114 | AGAGGCC**G**CCGAGGG |
|  |  |  | AGAGGCC**A**CCGAGGG |
|  | -326 | rs36211753 | TCTGATTT**T**ACGGAGAA |
|  |  |  | TCTGATTT**A**ACGGAGAA |
|  | -455 | rs36212119 | TAATTAA**A**AAGAAAAGC |
|  |  |  | TAATTAA**G**AAGAAAAGC |
|  | -548 | rs36212118 | CCAAGTA**C**TGTGCGA |
|  |  |  | CCAAGTA**T**TGTGCGA |
|  | -599 | rs36212117 | AGCTCTC**G**GCCAAGG |
|  |  |  | AGCTCTC**A**GCCAAGG |
|  | -786 G/A | rs36212115 | GGAGG**T**AG**G**TTTTGG |
|  |  |  | GGAGG**C**AG**A**TTTTGG |
|  | -783 G/A | rs36212116 | GGAGG**C**AG**G**TTTTGG |
|  |  |  | GGAGG**T**AG**A**TTTTGG |
|  | -905 | rs34304672 | CAAAAAAAAAAAA**C**AAAAAAAAAAC |
|  |  |  | CAAAAAAAAAAAA**A**AAAAAAAAAAC |
|  | -1140 | rs36212122 | GGTGGGC**G**GATCCTG |
|  |  |  | GGTGGGC**A**GATCCTG |
|  | -1193 | rs36212121 | CTAGGCC**G**GGCGCTG |
|  |  |  | CTAGGCC**A**GGCGCTG |
|  | -1269 | rs1741981 | GGAAGCT**A**TTCTGTG |
|  |  |  | GGAAGCT**G**TTCTGTG |
|  | -1546 | Applied for | GATAATTGAT**G**ATCTGG |
|  |  |  | GATAATTGAT**T**ATCTGG |
| **HTR2A** | -93 | rs7997977 | AAACCAA**A**CAGTGGA |
|  |  |  | AAACCAA**G**CAGTGGA |
|  | -119 G/C | rs36212789 | CACCAAA**GC**GAGGAC |
|  |  |  | CACCAAA**CA**GAGGAC |
|  | -120 C/A | rs7996679 | CACCAAA**GA**GAGGAC |
|  |  |  | CACCAAA**CC**GAGGAC |
|  | -179 | rs6310 | TTGAGCG**C**TCGGGAA |
|  |  |  | TTGAGCG**T**TCGGGAA |
|  | -268 | rs6307 | GTTATGC**C**GATGGTA |
|  |  |  | GTTATGC**T**GATGGTA |
|  | -427 | rs6309 | ATTCAGC**T**TAAGAATGG |
|  |  |  | ATTCAGC**C**TAAGAATGG |
|  | -651 | rs6312 | ATGTGTG**G**ACCCTGA |
|  |  |  | ATGTGTG**A**ACCCTGA |
|  | -850 | rs36212791 | GAGCC**G**TGCCAGAG |
|  |  |  | GAGCC**C**TGCCAGAG |
|  | -856 | rs36212792 | AACCCAGC**C**GAGCC |
|  |  |  | AACCCAGC**G**GAGCC |
|  | -1050 | rs6316 | CATACAC**A**TAGAGGG |
|  |  |  | CATACAC**G**TAGAGGG |
|  | -1305 | rs6311 | AAGTGCC**G**GACACTC |
|  |  |  | AAGTGCC**A**GACACTC |
|  | -1545 | rs17289304 | GGGAAAA**A**AATTGGT |
|  |  |  | GGGAAAA**C**AATTGGT |
|  | -1625 | rs36213156 | AGAGATC**G**AACTGCC |
|  |  |  | AGAGATC**A**AACTGCC |
|  | -1717 | rs1328685 | GAATTTGTC**C**AGGGAGA |
|  |  |  | GAATTTGTC**T**AGGGAGA |
|  | -1777 | rs36212784 | ATAATCAG**C**TATGAAGG |
|  |  |  | ATAATCAG**A**TATGAAGG |
|  | -1985 | rs36212451 | TGGCAGC**G**AGAATGA |
|  |  |  | TGGCAGC**A**AGAATGA |
|  | -2093 | rs731245 | CAGGAAA**T**AGGTGGC |
|  |  |  | CAGGAAA**C**AGGTGGC |
|  | -2131 | rs731244 | GCAGGCA**T**GGCAGTG |
|  |  |  | GCAGGCA**G**GGCAGTG |
| **IL1A** | -913 | rs1800587 | AGGCAACA**C**CATTGAAG |
|  |  |  | AGGCAACA**T**CATTGAAG |
|  | -1226 | rs1800794 | GAGGAAA**G**CAGCTTG |
|  |  |  | GAGGAAA**A**CAGCTTG |
|  | -1530 | rs3783521 | AGAGGAA**T**CAAGGTA |
|  |  |  | AGAGGAA**C**CAAGGTA |
| **MICA** | 60 | rs2844519 | GAAGCAG**G**AAGACCG |
|  |  |  | GAAGCAG**A**AAGACCG |
|  | -16 | rs2301749 | AGAAGGCG**C**CGCGGAAA |
|  |  |  | AGAAGGCG**T**CGCGGAAA |
|  | -29 | rs2301750 | GAAACTTA**G**TCACCTG |
|  |  |  | GAAACTTA**C**TCACCTG |
|  | -270 | rs6906175 | AAGCACGG**C**CAGGGC |
|  |  |  | AAGCACGG**G**CAGGGC |
|  | -284 | rs36222985 | GGAGGG**C**AGGCAGG |
|  |  |  | GGAGGG**T**AGGCAGG |
|  | -326 | rs36222984 | CCCACCC**G**GAGGAGC |
|  |  |  | CCCACCC**C**GAGGAGC |
|  |  |  | CCCACCC**T**GAGGAGC |
|  | -366 | rs36222682 | GGTTTAATGG**G**GCGGC |
|  |  |  | GGTTTAATGG**C**GCGGC |
|  | -418 | rs36222680 | GAGAATAG**C**CACGCGTT |
|  |  |  | GAGAATAG**T**CACGCGTT |
|  | -649 | rs36223345 | TCAGACA**C**TTAGAGG |
|  |  |  | TCAGACA**T**TTAGAGG |
|  | -708 | rs36223344 | GGGCCAA**G**GAGGCGG |
|  |  |  | GGGCCAA**A**GAGGCGG |
|  | -1004 | rs3763288 | CTGAAGG**G**AGGGGAA |
|  |  |  | CTGAAGG**A**AGGGGAA |
|  | -1224 | rs9266800 | AGATCCC**A**GAGCCCA |
|  |  |  | AGATCCC**C**GAGCCCA |
|  | -1364 | rs2596537 | AGTTGTA**A**ACACTGC |
|  |  |  | AGTTGTA**C**ACACTGC |
|  | -1483 | rs36223710 | GGAA**G**ATTTTTTTTCC |
|  |  |  | GGAA**T**ATTTTTTTTCC |
|  | -1709 | rs13220896 | AGCCACTG**C**GTAGAACC |
|  |  |  | AGCCACTG**T**GTAGAACC |
| **RB1** | -83 | rs36230211 | CTCGCGGACGTGACGGGA |
|  |  |  | GCCCGGGAGCGGA |
|  | -595 | rs3092875 | GAACGGA**T**AGTAGTG |
|  |  |  | GAACGGA**G**AGTAGTG |
|  | -768 | rs7337056 | TTAATC**C**CCAGAAAC |
|  |  |  | TTAATC**A**CCAGAAAC |
|  | -1554 | rs1573601 | GGTAGGA**C**TGGCTGT |
|  |  |  | GGTAGGA**A**TGGCTGT |
|  | -1733 | rs3092879 | CCAGCAGACTACTCC |
|  |  |  | CCAGCCTGATAAGAC |
| **SKP2** | -758 | rs36232788 | AGGGCATG**C**GCAGCCTT |
|  |  |  | AGGGCATG**G**GCAGCCTT |
|  | -846 | rs36232789 | ACCCGTGC**A**CGCAG |
|  |  |  | ACCCGTGC**G**CGCAG |
| **SMAD3** | -669 | rs11633026 | GCGACCC**G**GGCGGCG |
|  |  |  | GCGACCC**A**GGCGGCG |
|  | -749 | rs36222034 | GGAAGTC**C**TGGGAAA |
|  |  |  | GGAAGTC**T**TGGGAAA |
|  | -1137 | rs36221699 | TGGGGAC**A**CCAGTGA |
|  |  |  | TGGGGAC**C**CCAGTGA |
|  | -1938 | rs36221701 | CAGCGCGT**T**AACAGGAG |
|  |  |  | CAGCGCGT**C**AACAGGAG |
|  | -1946 | rs36221700 | ACAGCGG**C**AGCGCGT |
|  |  |  | ACAGCGG**A**AGCGCGT |
|  |  |  | GCCTTC**C**ACAGCCAGTCG |
| **SMAD4** | -412 | rs36222036 | CAAAAATTG**G**CCTTCAA |
|  |  |  | CAAAAATTG**T**CCTTCAA |
|  | -418F | rs36222037 | GCCTTC**A**ACAGCCAGTCG |
| **TFDP1** | -1394 | rs9603847 | TGTGGAC**A**CACGGAG |
|  |  |  | TGTGGAC**G**CACGGAG |
|  | -1515 | rs36203751 | GGGCACC**G**AGGACG |
|  |  |  | GGGCACT**T**AGGACG |
|  | -1516 | rs36203750 | GGGCAC**C**GAGGACG |
|  |  |  | GGGCAC**T**TAGGACG |
|  | -1530 | rs36203748 | CAGGCTG**C**GTCCGGT |
|  |  |  | CAGGCTG**T**GTCCGGT |
|  | -1655 | rs36204085 | GGCCCTA**G**CGGCGG |
|  |  |  | GGCCCTA**A**CGGCGG |
|  | -1779 | rs36204084 | GAGGACT**T**GAGGAGC |
|  |  |  | GAGGACT**C**GAGGAGC |
|  | -1883 | rs7323179 | GTGCGGC**G**TTGCCTG |
|  |  |  | GTGCGGC**C**TTGCCTG |
|  | -1887 | rs7324599 | GTG**C**GGCGTTGCCTG |
|  |  |  | GTG**T**GGCGTTGCCTG |
| **TGFB1** | -448 | rs11466314 | AGAGGGT**C**TGTCAAC |
|  |  |  | AGAGGGT**T**TGTCAAC |
|  | -508 | rs1800469 | CACCTGA**A**GGATGGA |
|  |  |  | CACCTGA**G**GGATGGA |
|  | -799 | rs1800468 | GTGGTGA**C**GTTGGAG |
|  |  |  | GTGGTGA**T**GTTGGAG |
|  | -1146 | rs3087453 | AAAGGAGA**G**AGAGGGGA |
|  |  |  | AAAGGAGA**C**AGAGGGGA |
|  | -1349 | rs36204367 | CCTTAGCA-GGGGAGTA |
|  |  |  | CCTTAGCA**G**GGGGAGTA |
|  | -1550 | rs11466313 | CATGAGG**---**GAAGGCA |
|  |  |  | CATGAGG**AGG**GAAGGCA |
|  | -1571 | rs4803457 | CCCAAAG**A**GAGCAGG |
|  |  |  | CCCAAAG**G**GAGCAGG |
|  | -1886 | rs2317130 | GGTAGAT**C**AGATGGT |
|  |  |  | GGTAGAT**T**AGATGGT |
|  | -1960 | rs11466311 | GAAG**G**GGATCAAGGA |
|  |  |  | GAAG**A**GGATCAAGGA |

**Supplementary Table S3. Description of haplotypes and their population frequencies**

Note that haplotypes were phased *in silico* by PHASE software, thus the rare ones and those with singleton mutations should be considered tentative. However, in principle this uncertainty in the structure of the rare haplotypes does not affect haplotype diversity indices.

**INDEX**

**AFR:** African-Americans and Sub-Saharan Africans

**AME:** Native Americans

**ASI:** South Eastern and Eastern Asians

**EUR:** Europeans

**MEA:** Middle East

**TOT:** Total sample

| BTN3A2 | Positions | | | | | | | | | | | | | | Counts | | | | | |
| --- | --- | --- | --- | --- | --- | --- | --- | --- | --- | --- | --- | --- | --- | --- | --- | --- | --- | --- | --- | --- |
|  | -1771 | -1733 | -1494 | -1403 | -1002 | -939 | -812 | -529 | -479 | -321 | -312 | -283 | -133 | -113 | AFR | AME | ASI | EUR | MEA | TOT |
| **Haplotype** |  |  |  |  |  |  |  |  |  |  |  |  |  |  |  |  |  |  |  |  |
| **Ancestral** | **T** | **C** | **-** | **T** | **G** | **C** | **A** | **C** | **G** | **A** | **T** | **A** | **C** | **C** |  |  |  |  |  |  |
| BT01 | C | - | - | C | - | - | - | T | - | - | C | - | - | G | 8 | 9 | 13 | 8 | 12 | 50 |
| BT02 | - | - | - | - | - | - | - | T | - | - | - | - | - | - | 3 | 3 | 0 | 3 | 2 | 11 |
| BT03 | - | - | - | - | - | - | - | - | - | - | - | - | - | - | 0 | 0 | 3 | 3 | 0 | 6 |
| BT04 | C | - | - | - | - | T | - | T | - | - | - | - | - | - | 0 | 2 | 0 | 1 | 1 | 4 |
| BT05 | - | T | - | - | - | - | - | T | T | - | - | - | - | - | 0 | 0 | 0 | 0 | 1 | 1 |
| BT07 | - | - | - | - | - | - | - | T | - | T | - | - | G | - | 2 | 0 | 0 | 0 | 0 | 2 |
| BT08 | C | - | INS A | C | - | - | - | T | - | - | C | - | - | G | 0 | 2 | 0 | 0 | 0 | 2 |
| BT11 | C | - | - | C | - | - | G | T | - | - | C | G | - | G | 0 | 0 | 0 | 1 | 0 | 1 |
| BT13 | C | - | - | C | T | - | - | T | - | - | C | - | - | G | 1 | 0 | 0 | 0 | 0 | 1 |
|  |  |  |  |  |  |  |  |  |  |  |  |  |  | total: | 14 | 16 | 16 | 16 | 16 | 78 |

| CAT | Positions | | | | | | | Counts | | | | | |
| --- | --- | --- | --- | --- | --- | --- | --- | --- | --- | --- | --- | --- | --- |
|  | -1632 | -1099 | -975 | -776 | -465 | -262 | -21 | AFR | AME | ASI | EUR | MEA | TOT |
| **Haplotype** |  |  |  |  |  |  |  |  |  |  |  |  |  |
| **Ancestral** | **C** | **T** | **T** | **A** | **G** | **C** | **G** |  |  |  |  |  |  |
| CA01 | - | - | - | - | - | - | T | 8 | 7 | 5 | 7 | 8 | 35 |
| CA02 | - | - | - | G | - | - | A | 2 | 7 | 7 | 0 | 1 | 17 |
| CA03 | - | - | - | - | - | T | T | 0 | 0 | 0 | 4 | 3 | 7 |
| CA04 | T | - | - | G | - | - | A | 1 | 1 | 3 | 3 | 4 | 12 |
| CA05 | T | - | C | - | - | - | T | 3 | 1 | 0 | 1 | 0 | 5 |
| CA07 | - | - | - | - | A | - | A | 2 | 0 | 0 | 0 | 0 | 2 |
| CA09 | - | - | - | G | - | - | T | 0 | 0 | 1 | 0 | 0 | 1 |
| CA10 | - | C | - | - | - | - | T | 0 | 0 | 0 | 1 | 0 | 1 |
|  |  |  |  |  |  |  | total: | 16 | 16 | 16 | 16 | 16 | 80 |

| CCND1 | Positions | | | | | | | | | | | | | Counts | | | | | |
| --- | --- | --- | --- | --- | --- | --- | --- | --- | --- | --- | --- | --- | --- | --- | --- | --- | --- | --- | --- |
|  | -1938 | -1931 | -1926 | -1988 | -1985 | -1737 | -1701 | -1597 | -1554 | -1537 | -766 | -568 | -519 | AFR | AME | ASI | EUR | MEA | TOT |
| **Haplotype** |  |  |  |  |  |  |  |  |  |  |  |  |  |  |  |  |  |  |  |
| **Ancestral** | **C** | **G** | **-** | **A** | **G** | **A** | **G** | **G** | **G** | **DEL C** | **A** | **A** | **C** |  |  |  |  |  |  |
| CCN1 | T | - | - | - | - | - | - | - | - | - | - | - | - | 6 | 12 | 12 | 7 | 8 | 45 |
| CCN2 | T | - | - | - | - | - | - | - | - | - | - | - | T | 2 | 0 | 0 | 0 | 0 | 2 |
| CCN3 | - | - | - | - | - | - | - | A | - | - | T | C | - | 1 | 0 | 0 | 0 | 0 | 1 |
| CCN6 | T | - | - | - | T | - | - | - | - | - | - | - | - | 0 | 0 | 1 | 0 | 0 | 1 |
| CCN7 | T | - | INS G | - | - | - | - | - | - | - | T | - | - | 2 | 0 | 0 | 0 | 0 | 2 |
| CCN8 | T | C | - | - | - | - | - | - | - | - | - | - | - | 0 | 0 | 0 | 1 | 1 | 2 |
| CCN10 | - | - | - | - | - | - | - | - | - | C | - | - | - | 3 | 3 | 3 | 6 | 5 | 20 |
| CCN11 | - | - | - | - | - | - | - | A | - | - | - | - | - | 1 | 0 | 0 | 0 | 0 | 1 |
| CCN12 | - | - | - | - | - | - | C | - | A | - | - | - | - | 1 | 0 | 0 | 0 | 1 | 2 |
| CCN13 | - | - | - | - | - | T | - | - | - | C | - | - | - | 0 | 0 | 0 | 1 | 0 | 1 |
| CCN14 | - | - | - | C | - | - | - | A | - | - | - | - | - | 0 | 1 | 0 | 1 | 1 | 3 |
|  |  |  |  |  |  |  |  |  |  |  |  |  | total: | 16 | 16 | 16 | 16 | 16 | 80 |

| CCNE1 | Positions | | Counts | | | | | |
| --- | --- | --- | --- | --- | --- | --- | --- | --- |
|  | -1938 | -1931 | AFR | AME | ASI | EUR | MEA | TOT |
| **Haplotype** |  |  |  |  |  |  |  |  |
| **Ancestral** | **T** | **G** |  |  |  |  |  |  |
| CCNE01 | - | - | 15 | 15 | 12 | 16 | 16 | 74 |
| CCNE02 | - | A | 0 | 1 | 2 | 0 | 0 | 3 |
| CCNE03 | DEL | - | 1 | 0 | 0 | 0 | 0 | 1 |
|  |  | total: | 16 | 16 | 14 | 16 | 16 | 78 |

| CDC25A | Positions | | | | | | | Counts | | | | | |
| --- | --- | --- | --- | --- | --- | --- | --- | --- | --- | --- | --- | --- | --- |
|  | -2030 | -1566 | -1440 | -1433 | -351 | -171 | -51 | AFR | AME | ASI | EUR | MEA | TOT |
| **Haplotype** |  |  |  |  |  |  |  |  |  |  |  |  |  |
| **Ancestral** | **G** | **C** | **G** | **C** | **G** | **A** | **G** |  |  |  |  |  |  |
| CDC1 | - | - | - | - | - | - | - | 13 | 13 | 13 | 12 | 14 | 65 |
| CDC2 | - | - | - | - | - | - | C | 0 | 2 | 2 | 0 | 0 | 4 |
| CDC3 | - | - | - | - | - | T | - | 0 | 0 | 1 | 0 | 1 | 2 |
| CDC4 | - | - | - | - | A | - | - | 0 | 1 | 0 | 0 | 0 | 1 |
| CDC5 | - | - | A | - | - | - | - | 2 | 0 | 0 | 0 | 0 | 2 |
| CDC6 | - | T | - | T | - | - | - | 1 | 0 | 0 | 0 | 0 | 1 |
| CDC7 | T | - | - | - | - | - | - | 0 | 0 | 0 | 4 | 1 | 5 |
|  |  |  |  |  |  |  | total: | 16 | 16 | 16 | 16 | 16 | 80 |

| CDKN1a | Positions | | | | | | | | | | | Counts | | | | | |
| --- | --- | --- | --- | --- | --- | --- | --- | --- | --- | --- | --- | --- | --- | --- | --- | --- | --- |
|  | -1731 | -1536 | -1529 | -1466 | -1284 | -1021 | -899 | -791 | -519 | -499 | -134 | AFR | AME | ASI | EUR | MEA | TOT |
| **Haplotype** |  |  |  |  |  |  |  |  |  |  |  |  |  |  |  |  |  |
| **Ancestral** | **T** | **C** | **T** | **G** | **T** | **G** | **A** | **G** | **G** | **G** | **G** |  |  |  |  |  |  |
| CKa1 | - | - | - | A | - | - | - | A | - | - | - | 4 | 7 | 5 | 11 | 10 | 37 |
| CKa2 | - | - | - | A | - | - | C | A | - | - | - | 0 | 1 | 3 | 0 | 0 | 4 |
| CKa3 | - | - | - | A | - | - | C | - | - | - | - | 3 | 2 | 2 | 0 | 1 | 8 |
| CKa4 | - | - | - | A | - | - | C | - | C | C | - | 1 | 0 | 0 | 0 | 0 | 1 |
| CKa6 | - | - | - | A | C | - | - | - | - | - | - | 1 | 0 | 0 | 0 | 0 | 1 |
| CKa7 | - | - | - | A | C | - | C | A | - | - | - | 0 | 1 | 0 | 0 | 0 | 1 |
| CKa8 | - | - | - | A | C | - | C | - | - | - | - | 1 | 1 | 0 | 0 | 0 | 2 |
| CKa9 | - | - | - | A | C | - | C | - | C | C | - | 0 | 1 | 3 | 2 | 2 | 8 |
| CKa10 | - | - | - | A | C | A | - | - | - | - | C | 0 | 2 | 0 | 0 | 0 | 2 |
| CKa12 | - | - | C | - | C | - | - | - | - | - | - | 5 | 0 | 0 | 0 | 0 | 5 |
| CKa15 | - | - | C | - | C | A | - | - | - | - | C | 0 | 0 | 0 | 1 | 0 | 1 |
| CKa16 | - | T | C | - | C | A | - | - | - | - | C | 0 | 0 | 3 | 2 | 3 | 8 |
| CKa17 | DEL | - | - | A | C | - | - | - | C | C | - | 1 | 1 | 0 | 0 | 0 | 2 |
|  |  |  |  |  |  |  |  |  |  |  | total: | 16 | 16 | 16 | 16 | 16 | 80 |

| CDKN1b | Positions | | | | | | | | | | Counts | | | | | |
| --- | --- | --- | --- | --- | --- | --- | --- | --- | --- | --- | --- | --- | --- | --- | --- | --- |
|  | -1908 | -1857 | -1608 | -1228 | -1084 | -756 | -522 | -373 | -337 | -221 | AFR | AME | ASI | EUR | MEA | TOT |
| **Haplotype** |  |  |  |  |  |  |  |  |  |  |  |  |  |  |  |  |
| **Ancestral** | **T** | **C** | **C** | **C** | **CTT** | **T** | **C** | **C** | **T** | **T** |  |  |  |  |  |  |
| CKb1 | - | - | - | - | - | - | - | - | - | - | 1 | 4 | 11 | 6 | 3 | 25 |
| CKb2 | - | - | - | - | - | - | - | - | G | - | 1 | 0 | 0 | 0 | 1 | 2 |
| CKb3 | - | - | - | - | - | - | - | A | - | - | 2 | 5 | 5 | 8 | 6 | 26 |
| CKb4 | - | - | - | - | - | - | T | - | G | - | 4 | 2 | 0 | 2 | 0 | 8 |
| CKb5 | - | T | - | - | - | - | - | - | G | C | 1 | 0 | 0 | 0 | 0 | 1 |
| CKb7 | - | - | T | - | - | - | - | - | - | - | 2 | 0 | 0 | 0 | 0 | 2 |
| CKb8 | - | - | T | - | - | G | - | - | - | - | 0 | 2 | 0 | 0 | 1 | 3 |
| CKb9 | - | T | - | - | - | - | - | - | G | - | 2 | 3 | 0 | 0 | 5 | 10 |
| CKb10 | DEL | - | - | - | - | - | - | - | - | - | 1 | 0 | 0 | 0 | 0 | 1 |
| CKb11 | DEL | - | - | T | - | - | - | - | - | - | 1 | 0 | 0 | 0 | 0 | 1 |
| CKb12 | DEL | - | - | - | DEL | - | - | - | - | - | 1 | 0 | 0 | 0 | 0 | 1 |
|  |  |  |  |  |  |  |  |  |  | total: | 16 | 16 | 16 | 16 | 16 | 80 |

| CDKN2a | Positions | | | | | | | | Counts | | | | | |
| --- | --- | --- | --- | --- | --- | --- | --- | --- | --- | --- | --- | --- | --- | --- |
|  | -1879 | -1600 | -710 | -598 | -589 | -464 | -222 | -44 | AFR | AME | ASI | EUR | MEA | TOT |
| **Haplotype** |  |  |  |  |  |  |  |  |  |  |  |  |  |  |
| **Ancestral** | **G** | **A** | **C** | **G** | **G** | **C** | **T** | **A** |  |  |  |  |  |  |
| CDKN2A01 | - | - | - | - | - | - | - | - | 15 | 10 | 12 | 14 | 13 | 64 |
| CDKN2A02 | - | - | - | - | - | - | A | - | 0 | 4 | 0 | 2 | 0 | 6 |
| CDKN2A03 | - | - | - | - | - | T | - | - | 0 | 1 | 0 | 0 | 1 | 2 |
| CDKN2A04 | - | - | - | A | DEL | - | - | T | 0 | 0 | 4 | 0 | 0 | 4 |
| CDKN2A05 | - | - | A | - | - | - | - | - | 0 | 1 | 0 | 0 | 1 | 2 |
| CDKN2A06 | - | G | - | - | - | - | - | - | 1 | 0 | 0 | 0 | 0 | 1 |
| CDKN2A07 | A | - | - | - | - | - | - | - | 0 | 0 | 0 | 0 | 1 | 1 |
|  |  |  |  |  |  |  |  | total: | 16 | 16 | 16 | 16 | 16 | 80 |

| CX3CR1 | Positions | | | | | | | | | | | | | | | | Counts | | | | | |
| --- | --- | --- | --- | --- | --- | --- | --- | --- | --- | --- | --- | --- | --- | --- | --- | --- | --- | --- | --- | --- | --- | --- |
|  | -1943 | -1683 | -1484 | -1469 | -1363 | -1346 | -1345 | -1242 | -1185 | -986 | -738 | -387 | -325 | -302 | -58 | -36 | AFR | AME | ASI | EUR | MEA | TOT |
| **Haplotype** |  |  |  |  |  |  |  |  |  |  |  |  |  |  |  |  |  |  |  |  |  |  |
| **Ancestral** | **C** | **A** | **G** | **G** | **G** | **G** | **C** | **C** | **A** | **C** | **C** | **C** | **T** | **G** | **C** | **G** |  |  |  |  |  |  |
| CX1 | G | C | C | - | - | T | - | - | G | - | - | T | - | - | - | - | 0 | 10 | 6 | 5 | 2 | 23 |
| CX2 | - | - | C | - | C | - | T | - | G | T | - | - | - | - | - | - | 2 | 1 | 5 | 4 | 2 | 14 |
| CX3 | - | - | C | - | - | - | - | - | G | - | - | - | - | - | - | - | 1 | 3 | 0 | 1 | 2 | 7 |
| CX4 | - | C | C | - | C | - | - | - | G | - | - | - | C | - | - | - | 1 | 2 | 0 | 2 | 6 | 11 |
| CX5 | - | - | C | - | - | - | - | - | - | - | - | - | - | - | - | - | 3 | 0 | 1 | 1 | 1 | 6 |
| CX6 | G | C | C | - | - | T | - | - | G | - | - | - | - | - | - | - | 0 | 0 | 4 | 0 | 0 | 4 |
| CX7 | - | C | C | - | - | T | - | - | G | - | - | T | - | - | - | - | 0 | 0 | 0 | 0 | 1 | 1 |
| CX8 | - | - | C | - | - | - | - | - | - | - | - | - | - | - | - | A | 0 | 0 | 0 | 1 | 1 | 2 |
| CX9 | - | - | T | - | C | - | T | - | G | T | - | - | - | - | - | - | 0 | 0 | 0 | 1 | 0 | 1 |
| CX10 | - | - | C | - | C | - | - | - | G | - | - | - | - | - | - | - | 1 | 0 | 0 | 0 | 0 | 1 |
| CX11 | - | - | - | T | - | T | - | - | G | T | - | T | - | - | - | - | 3 | 0 | 0 | 0 | 0 | 3 |
| CX12 | - | - | C | - | C | - | T | T | G | T | - | - | - | - | - | - | 2 | 0 | 0 | 0 | 0 | 2 |
| CX16 | - | - | C | - | C | - | T | T | G | T | - | - | - | - | A | - | 1 | 0 | 0 | 0 | 0 | 1 |
| CX17 | - | - | C | - | - | - | T | - | - | - | - | - | - | - | - | - | 0 | 0 | 0 | 0 | 1 | 1 |
| CX19 | - | - | C | - | - | T | - | - | - | T | - | - | - | - | - | - | 1 | 0 | 0 | 0 | 0 | 1 |
| CX20 | - | - | C | - | - | T | - | - | - | T | T | - | - | - | - | - | 1 | 0 | 0 | 0 | 0 | 1 |
| CX28 | G | C | C | - | - | T | - | - | G | - | - | T | - | A | - | - | 0 | 0 | 0 | 1 | 0 | 1 |
|  |  |  |  |  |  |  |  |  |  |  |  |  |  |  |  | total: | 16 | 16 | 16 | 16 | 16 | 80 |

| E2F1 | Positions | | | | Counts | | | | | |
| --- | --- | --- | --- | --- | --- | --- | --- | --- | --- | --- |
|  | -897 | -300 | -243 | -187 | AFR | AME | ASI | EUR | MEA | TOT |
| **Haplotype** |  |  |  |  |  |  |  |  |  |  |
| **Ancestral** | **C** | **C** | **A** | **G** |  |  |  |  |  |  |
| E2F01 | - | - | - | - | 13 | 13 | 9 | 13 | 11 | 59 |
| E2F02 | - | - | - | A | 3 | 3 | 3 | 3 | 2 | 14 |
| E2F03 | T | - | - | - | 0 | 0 | 2 | 0 | 3 | 5 |
| E2F04 | - | - | C | A | 0 | 0 | 1 | 0 | 0 | 1 |
| E2F05 | - | T | - | - | 0 | 0 | 1 | 0 | 0 | 1 |
|  |  |  |  | total: | 16 | 16 | 16 | 16 | 16 | 80 |

| FEN1 | Positions | | | | | Counts | | | | | |
| --- | --- | --- | --- | --- | --- | --- | --- | --- | --- | --- | --- |
|  | -1804 | -1733 | -1688 | -704 | -69 | AFR | AME | ASI | EUR | MEA | TOT |
| **Haplotype** |  |  |  |  |  |  |  |  |  |  |  |
| **Ancestral** | **T** | **TTT** | **C** | **C** | **G** |  |  |  |  |  |  |
| FE01 | - | - | - | - | - | 11 | 7 | 5 | 13 | 9 | 45 |
| FE02 | - | - | - | - | A | 2 | 8 | 10 | 3 | 7 | 30 |
| FE03 | - | - | - | T | - | 2 | 0 | 0 | 0 | 0 | 2 |
| FE04 | - | DEL | - | - | A | 0 | 0 | 1 | 0 | 0 | 1 |
| FE05 | - | - | T | - | - | 0 | 1 | 0 | 0 | 0 | 1 |
| FE06 | C | - | - | - | - | 1 | 0 | 0 | 0 | 0 | 1 |
|  |  |  |  |  | total: | 16 | 16 | 16 | 16 | 16 | 80 |

| FGB | Positions | | | | | | | | | | | | | Counts | | | | | |
| --- | --- | --- | --- | --- | --- | --- | --- | --- | --- | --- | --- | --- | --- | --- | --- | --- | --- | --- | --- |
|  | -1852 | -1832 | -1552 | -1420 | -1143 | -993 | -854 | -849 | -727 | -455 | -249 | -199 | -148 | AFR | AME | ASI | EUR | MEA | TOT |
| **Haplotype** |  |  |  |  |  |  |  |  |  |  |  |  |  |  |  |  |  |  |  |
| **Ancestral** | **A** | **G** | **A** | **G** | **A** | **C** | **G** | **G** | **A** | **G** | **C** | **G** | **T** |  |  |  |  |  |  |
| FG01 | - | - | - | - | - | - | - | - | - | - | - | - | C | 6 | 9 | 3 | 6 | 2 | 26 |
| FG02 | - | - | - | - | - | - | - | - | - | - | T | - | C | 2 | 2 | 9 | 2 | 4 | 19 |
| FG03 | - | - | - | A | - | T | - | - | - | A | - | - | - | 1 | 0 | 2 | 5 | 5 | 13 |
| FG04 | - | - | - | - | - | - | A | - | - | - | - | - | C | 0 | 5 | 1 | 2 | 4 | 12 |
| FG05 | - | - | - | - | G | - | - | - | - | - | - | - | C | 2 | 0 | 0 | 0 | 0 | 2 |
| FG06 | G | - | - | - | G | - | - | - | - | - | - | - | C | 3 | 0 | 0 | 0 | 0 | 3 |
| FG07 | - | - | G | - | - | - | - | - | - | - | - | - | C | 1 | 0 | 0 | 0 | 0 | 1 |
| FG09 | - | A | - | - | - | - | - | - | - | - | T | - | C | 0 | 0 | 1 | 0 | 0 | 1 |
| FG10 | - | - | - | - | - | - | - | - | T | - | - | - | C | 0 | 0 | 0 | 0 | 1 | 1 |
| FG11 | - | - | - | - | - | - | - | - | - | - | - | A | C | 1 | 0 | 0 | 0 | 0 | 1 |
| FG12 | - | - | - | - | - | - | - | C | - | - | T | - | C | 0 | 0 | 0 | 1 | 0 | 1 |
|  |  |  |  |  |  |  |  |  |  |  |  |  | total: | 16 | 16 | 16 | 16 | 16 | 80 |

| GPX2 | Positions | | | | | | | | | | | | | | | | Counts | | | | | |
| --- | --- | --- | --- | --- | --- | --- | --- | --- | --- | --- | --- | --- | --- | --- | --- | --- | --- | --- | --- | --- | --- | --- |
|  | -2077 | -2022 | -1913 | -1723 | -1659 | -1276 | -1195 | -1070 | -843 | -724 | -524 | -512 | -442 | -360 | -199 | -166 | AFR | AME | ASI | EUR | MEA | TOT |
| **HapName** |  |  |  |  |  |  |  |  |  |  |  |  |  |  |  |  |  |  |  |  |  |  |
| **Ancestral** | **A** | **T** | **G** | **C** | **T** | **T** | **C** | **T** | **A** | **T** | **T** | **C** | **A** | **A** | **C** | **A** |  |  |  |  |  |  |
| XII01 | - | - | - | - | - | C | - | C | - | - | - | - | - | - | - | - | 4 | 8 | 3 | 13 | 11 | 39 |
| XII02 | - | - | - | - | - | C | - | - | - | DEL | - | A | - | - | - | - | 1 | 0 | 9 | 0 | 1 | 11 |
| XII03 | - | - | - | T | - | C | - | - | - | DEL | C | - | - | - | - | - | 0 | 2 | 2 | 1 | 2 | 7 |
| XII04 | - | G | - | - | - | C | - | - | G | DEL | - | - | - | - | - | - | 2 | 0 | 0 | 1 | 0 | 3 |
| XII05 | - | - | - | - | - | C | - | C | - | DEL | - | - | - | - | - | - | 0 | 1 | 0 | 1 | 0 | 2 |
| XII06 | G | G | - | - | - | C | - | C | - | - | - | - | - | - | - | G | 2 | 2 | 0 | 0 | 0 | 4 |
| XII07 | - | - | - | - | - | C | - | - | - | - | - | A | - | - | - | - | 0 | 2 | 1 | 0 | 0 | 3 |
| XII08 | - | - | - | - | - | C | - | - | - | DEL | - | - | - | - | - | - | 1 | 1 | 0 | 0 | 0 | 2 |
| XII09 | - | - | - | - | - | C | - | - | - | - | - | - | - | - | - | - | 1 | 0 | 0 | 0 | 0 | 1 |
| XII10 | - | - | - | T | - | C | - | - | - | - | C | - | - | - | - | - | 0 | 0 | 0 | 0 | 1 | 1 |
| XII14 | - | - | - | - | - | C | - | C | - | - | - | - | - | G | - | - | 0 | 0 | 0 | 0 | 1 | 1 |
| XII17 | - | - | - | - | - | C | - | - | - | - | - | - | - | - | G | - | 1 | 0 | 0 | 0 | 0 | 1 |
| XII20 | - | - | - | - | - | C | - | C | - | - | - | - | - | - | - | G | 1 | 0 | 0 | 0 | 0 | 1 |
| XII21 | - | - | - | - | - | C | T | C | - | DEL | - | - | - | - | - | - | 1 | 0 | 0 | 0 | 0 | 1 |
| XII22 | - | - | - | - | - | - | - | - | - | DEL | - | - | - | - | - | - | 1 | 0 | 0 | 0 | 0 | 1 |
| XII24 | - | - | - | - | C | - | - | - | - | DEL | - | - | - | - | - | - | 1 | 0 | 0 | 0 | 0 | 1 |
| XII27 | - | - | A | - | - | C | - | - | - | DEL | - | A | G | - | - | - | 0 | 0 | 1 | 0 | 0 | 1 |
|  |  |  |  |  |  |  |  |  |  |  |  |  |  |  |  | total: | 16 | 16 | 16 | 16 | 16 | 80 |

| GPX3 | Positions | | | | | | | | | | | | Counts | | | | | |
| --- | --- | --- | --- | --- | --- | --- | --- | --- | --- | --- | --- | --- | --- | --- | --- | --- | --- | --- |
|  | -2005 | -1958 | -776 | -709 | -694 | -628 | -617 | -335 | -285 | -80 | -69 | -51 | AFR | AME | ASI | EUR | MEA | TOT |
| **HapName** |  |  |  |  |  |  |  |  |  |  |  |  |  |  |  |  |  |  |
| **Ancestral** | **T** | **C** | **C** | **A** | **C** | **A** | **C** | **C** | **C** | **C** | **T** | **T** |  |  |  |  |  |  |
| XIII01 | C | T | - | - | T | - | - | T | T | - | A | - | 4 | 10 | 11 | 9 | 9 | 43 |
| XIII02 | - | - | - | - | T | - | - | T | T | - | A | - | 2 | 1 | 0 | 2 | 5 | 10 |
| XIII03 | C | - | - | C | - | T | - | - | - | - | - | - | 3 | 2 | 1 | 1 | 0 | 7 |
| XIII04 | - | - | - | - | T | - | - | T | T | - | A | A | 3 | 0 | 3 | 0 | 0 | 6 |
| XIII05 | C | - | - | - | T | - | - | T | T | - | A | - | 1 | 1 | 0 | 0 | 0 | 2 |
| XIII09 | C | - | - | C | - | - | - | - | - | - | - | - | 0 | 2 | 0 | 0 | 0 | 2 |
| XIII16 | C | - | - | C | - | T | - | T | - | - | - | - | 0 | 0 | 1 | 0 | 0 | 1 |
| XIII17 | C | - | - | C | - | T | - | - | - | - | A | - | 0 | 0 | 0 | 0 | 1 | 1 |
| XIII18 | C | - | - | C | - | T | T | - | - | - | - | - | 1 | 0 | 0 | 0 | 0 | 1 |
| XIII19 | C | - | T | - | T | - | - | T | T | - | A | - | 0 | 0 | 0 | 0 | 1 | 1 |
|  |  |  |  |  |  |  |  |  |  |  |  | total: | 14 | 16 | 16 | 12 | 16 | 74 |

| GSS | Positions | | | | | | | | | Counts | | | | | |
| --- | --- | --- | --- | --- | --- | --- | --- | --- | --- | --- | --- | --- | --- | --- | --- |
|  | -1979 | -1943 | -1762 | -1494 | -1412 | -1125 | -792 | -716 | -514 | AFR | AME | ASI | EUR | MEA | TOT |
| **HapName** |  |  |  |  |  |  |  |  |  |  |  |  |  |  |  |
| **Ancestral** | **G** | **G** | **G** | **G** | **A** | **C** | **G** | **C** | **G** |  |  |  |  |  |  |
| S1 | - | A | - | - | - | - | - | - | C | 6 | 7 | 8 | 8 | 6 | 35 |
| S2 | A | A | - | - | - | - | - | - | - | 1 | 5 | 5 | 5 | 3 | 19 |
| S3 | - | A | - | A | - | - | - | - | - | 0 | 3 | 0 | 3 | 6 | 12 |
| S4 | - | A | - | - | T | - | - | G | - | 2 | 1 | 2 | 0 | 0 | 5 |
| S5 | - | A | - | - | - | - | DEL | - | - | 3 | 0 | 0 | 0 | 0 | 3 |
| S6 | - | - | - | - | - | - | - | - | - | 2 | 0 | 0 | 0 | 0 | 2 |
| S7 | - | A | - | - | - | - | - | - | - | 2 | 0 | 0 | 0 | 0 | 2 |
| S8 | - | A | - | - | - | - | - | G | - | 0 | 0 | 0 | 0 | 0 | 0 |
| S9 | - | A | - | - | T | T | - | G | - | 0 | 0 | 0 | 0 | 1 | 1 |
| S10 | A | A | - | A | - | - | - | - | - | 0 | 0 | 0 | 0 | 0 | 0 |
| S11 | A | A | A | - | - | - | - | - | - | 0 | 0 | 1 | 0 | 0 | 1 |
|  |  |  |  |  |  |  |  |  | total: | 16 | 16 | 16 | 16 | 16 | 80 |

| GSTM3 | Positions | | | | | | Counts | | | | | |
| --- | --- | --- | --- | --- | --- | --- | --- | --- | --- | --- | --- | --- |
|  | -1286 | -859 | -208 | -46 | 14 | 17 | AFR | AME | ASI | EUR | MEA | TOT |
| **HapName** |  |  |  |  |  |  |  |  |  |  |  |  |
| **Ancestral** | **C** | **C** | **C** | **G** | **G** | **A** |  |  |  |  |  |  |
| MIII-1 | - | - | - | - | - | G | 0 | 6 | 2 | 7 | 8 | 23 |
| MIII-2 | - | T | - | T | - | G | 14 | 4 | 4 | 4 | 3 | 29 |
| MIII-3 | G | T | - | T | - | G | 0 | 6 | 10 | 4 | 4 | 24 |
| MIII-4 | - | - | - | - | - | - | 0 | 0 | 0 | 0 | 1 | 1 |
| MIII-6 | - | T | - | T | A | G | 1 | 0 | 0 | 0 | 0 | 1 |
| MIII-7 | - | T | G | T | - | G | 1 | 0 | 0 | 0 | 0 | 1 |
| MIII-8 | G | - | - | - | - | G | 0 | 0 | 0 | 1 | 0 | 1 |
|  |  |  |  |  |  | total: | 16 | 16 | 16 | 16 | 16 | 80 |

| GSTM4 | Positions | | | | | | | | | | Counts | | | | | |
| --- | --- | --- | --- | --- | --- | --- | --- | --- | --- | --- | --- | --- | --- | --- | --- | --- |
|  | -1617 | -1614 | -1302 | -1196 | -823 | -822 | -821 | -584 | -195 | -22 | AFR | AME | ASI | EUR | MEA | TOT |
| **HapName** |  |  |  |  |  |  |  |  |  |  |  |  |  |  |  |  |
| **Ancestral** | **A** | **G** | **C** | **C** | **T** | **T** | **C** | **T** | **C** | **G** |  |  |  |  |  |  |
| MIV-1 | G | A | - | - | - | - | - | - | - | C | 1 | 6 | 8 | 6 | 7 | 28 |
| MIV-2 | - | - | T | - | - | - | - | - | - | - | 5 | 5 | 7 | 7 | 4 | 28 |
| MIV-3 | - | A | - | - | - | - | - | - | - | - | 5 | 3 | 0 | 3 | 4 | 15 |
| MIV-4 | - | A | - | - | - | G | - | - | - | - | 0 | 1 | 0 | 0 | 0 | 1 |
| MIV-5 | - | - | - | - | - | - | - | - | - | - | 1 | 1 | 0 | 0 | 0 | 2 |
| MIV-6 | G | A | - | - | C | - | - | - | - | C | 2 | 0 | 0 | 0 | 0 | 2 |
| MIV-7 | - | A | - | G | - | - | - | C | G | - | 2 | 0 | 0 | 0 | 0 | 2 |
| MIV-8 | G | A | - | - | - | - | - | - | - | - | 0 | 0 | 1 | 0 | 0 | 1 |
| MIV-9 | - | A | - | - | - | - | T | - | - | - | 0 | 0 | 0 | 0 | 1 | 1 |
|  |  |  |  |  |  |  |  |  |  | total: | 16 | 16 | 16 | 16 | 16 | 80 |

| GSTP1 | Positions | | | | | | | | | |  |  |  | Counts | | | | | |
| --- | --- | --- | --- | --- | --- | --- | --- | --- | --- | --- | --- | --- | --- | --- | --- | --- | --- | --- | --- |
|  | -2088 | -1964 | -1937 | -1736 | -1386 | -1231 | -829 | -828 | -564 | -286 | -285 | -280 | -189 | AFR | AME | ASI | EUR | MEA | TOT |
| **HapName** |  |  |  |  |  |  |  |  |  |  |  |  |  |  |  |  |  |  |  |
| **Ancestral** | **INS a** | **C** | **G** | **A** | **A** | **C** | **G** | **C** | **G** | **DEL T** | **A** | **T** | **C** |  |  |  |  |  |  |
| TP01 | - | - | - | T | - | - | - | - | C | T | C | C | - | 1 | 5 | 10 | 11 | 10 | 37 |
| TP02 | - | T | A | - | G | - | - | - | - | - | - | - | - | 6 | 5 | 2 | 4 | 4 | 21 |
| TP03 | - | - | - | T | - | - | C | T | - | T | C | C | - | 1 | 4 | 1 | 0 | 2 | 8 |
| TP04 | DEL | - | - | T | - | - | - | - | - | T | C | C | G | 1 | 1 | 2 | 0 | 0 | 4 |
| TP06 | - | - | - | T | - | - | - | - | - | T | C | C | - | 2 | 0 | 0 | 0 | 0 | 2 |
| TP07 | - | - | A | T | - | - | - | - | C | T | C | C | - | 0 | 0 | 1 | 0 | 0 | 1 |
| TP09 | - | - | - | T | - | - | - | - | C | - | - | - | - | 0 | 1 | 0 | 0 | 0 | 1 |
| TP10 | - | - | - | T | - | - | C | T | C | T | C | C | - | 0 | 0 | 0 | 1 | 0 | 1 |
| TP11 | - | T | A | - | G | - | - | - | C | - | - | - | - | 1 | 0 | 0 | 0 | 0 | 1 |
| TP12 | DEL | - | - | T | - | - | - | - | - | T | C | C | - | 1 | 0 | 0 | 0 | 0 | 1 |
| TP13 | DEL | - | - | T | - | T | - | - | - | T | C | C | - | 2 | 0 | 0 | 0 | 0 | 2 |
| TP15 | - | - | - | T | - | T | - | - | - | T | C | C | - | 1 | 0 | 0 | 0 | 0 | 1 |
|  | INS a = INS AGTCTAGTCTAGT | | | | | | | | |  |  |  | total: | 16 | 16 | 16 | 16 | 16 | 80 |

| HDAC1 | Positions | | | | | | | | | | | | Counts | | | | | |
| --- | --- | --- | --- | --- | --- | --- | --- | --- | --- | --- | --- | --- | --- | --- | --- | --- | --- | --- |
|  | -1546 | -1269 | -1193 | -1140 | -905 | -786 | -783 | -599 | -548 | -455 | -326 | -162 | AFR | AME | ASI | EUR | MEA | TOT |
| **HapName** |  |  |  |  |  |  |  |  |  |  |  |  |  |  |  |  |  |  |
| **Ancestral** | **G** | **C** | **G** | **G** | **A** | **T** | **G** | **C** | **G** | **T** | **A** | **C** |  |  |  |  |  |  |
| HD01 | - | T | - | - | - | - | A | - | - | - | - | - | 1 | 12 | 6 | 8 | 10 | 37 |
| HD02 | - | - | - | - | - | - | A | - | - | - | - | - | 8 | 4 | 7 | 5 | 4 | 28 |
| HD03 | - | T | - | - | - | - | A | - | - | C | - | - | 0 | 0 | 0 | 2 | 2 | 4 |
| HD04 | - | - | - | - | - | - | - | T | A | - | T | - | 2 | 0 | 0 | 0 | 0 | 2 |
| HD05 | - | - | - | - | - | - | - | - | - | - | - | - | 2 | 0 | 0 | 0 | 0 | 2 |
| HD06 | - | - | - | A | - | - | A | - | - | - | - | - | 1 | 0 | 0 | 0 | 0 | 1 |
| HD07 | - | T | - | - | - | - | A | - | - | C | - | T | 0 | 0 | 0 | 1 | 0 | 1 |
| HD08 | T | - | - | - | - | - | A | - | - | - | - | - | 0 | 0 | 1 | 0 | 0 | 1 |
| HD09 | - | T | A | - | - | - | A | - | - | - | - | - | 0 | 0 | 2 | 0 | 0 | 2 |
| HD11 | - | - | - | - | - | C | A | - | - | - | - | - | 1 | 0 | 0 | 0 | 0 | 1 |
| HD12 | - | - | - | - | C | - | A | - | - | - | - | - | 1 | 0 | 0 | 0 | 0 | 1 |
|  |  |  |  |  |  |  |  |  |  |  |  | total: | 16 | 16 | 16 | 16 | 16 | 80 |

| HTR2A | Positions | | | | | | | | | | | | | | | | | | Counts | | | | | |
| --- | --- | --- | --- | --- | --- | --- | --- | --- | --- | --- | --- | --- | --- | --- | --- | --- | --- | --- | --- | --- | --- | --- | --- | --- |
|  | -2131 | -2093 | -1985 | -1777 | -1717 | -1625 | -1545 | -1305 | -1050 | -856 | -850 | -651 | -427 | -268 | -179 | -120 | -119 | -93 | AFR | AME | ASI | EUR | MEA | TOT |
| **HapName** |  |  |  |  |  |  |  |  |  |  |  |  |  |  |  |  |  |  |  |  |  |  |  |  |
| **Ancestral** | **C** | **A** | **C** | **G** | **A** | **C** | **T** | **C** | **T** | **G** | **C** | **G** | **G** | **C** | **C** | **C** | **G** | **T** |  |  |  |  |  |  |
| HT01 | - | G | - | - | - | - | - | T | - | - | - | A | - | - | T | - | C | - | 7 | 6 | 11 | 6 | 10 | 40 |
| HT02 | - | - | - | - | - | - | - | - | - | - | - | A | - | - | T | - | C | - | 1 | 4 | 2 | 5 | 4 | 16 |
| HT03 | - | G | - | - | - | - | - | - | - | - | - | A | - | - | T | - | C | - | 1 | 2 | 1 | 1 | 1 | 6 |
| HT04 | A | - | - | - | G | T | G | - | - | - | - | A | - | - | T | - | C | - | 1 | 2 | 1 | 1 | 0 | 5 |
| HT05 | A | - | - | - | G | T | G | - | - | - | - | - | - | - | - | - | C | - | 1 | 0 | 0 | 2 | 1 | 4 |
| HT06 | - | - | - | T | - | - | - | - | - | - | - | A | - | - | T | - | C | - | 0 | 2 | 0 | 0 | 0 | 2 |
| HT07 | - | G | T | - | - | - | - | - | - | - | - | A | - | - | T | A | C | - | 1 | 0 | 0 | 0 | 0 | 1 |
| HT08 | - | G | - | - | - | - | - | T | C | - | - | A | - | - | T | - | C | - | 1 | 0 | 0 | 0 | 0 | 1 |
| HT09 | - | G | - | - | - | - | - | - | - | - | - | A | - | - | - | - | - | - | 0 | 0 | 0 | 1 | 0 | 1 |
| HT10 | - | G | - | - | - | - | - | T | - | - | - | A | - | T | T | - | C | - | 1 | 0 | 0 | 0 | 0 | 1 |
| HT12 | A | - | - | - | G | - | - | - | - | - | - | - | A | - | - | - | C | - | 1 | 0 | 0 | 0 | 0 | 1 |
| HT18 | - | G | - | - | - | - | - | - | - | C | - | A | - | - | T | - | C | - | 0 | 0 | 1 | 0 | 0 | 1 |
| HT19 | - | G | T | - | - | - | - | - | - | - | G | A | - | - | T | A | C | C | 1 | 0 | 0 | 0 | 0 | 1 |
|  |  |  |  |  |  |  |  |  |  |  |  |  |  |  |  |  |  | total: | 16 | 16 | 16 | 16 | 16 | 80 |

| IL1A | Positions | | | Counts | | | | | |
| --- | --- | --- | --- | --- | --- | --- | --- | --- | --- |
|  | -1530 | -1226 | -913 | AFR | AME | ASI | EUR | MEA | TOT |
| **Haplotype** |  |  |  |  |  |  |  |  |  |
| **Ancestral** | **G** | **G** | **G** |  |  |  |  |  |  |
| IL1 | - | - | - | 9 | 7 | 4 | 7 | 9 | 36 |
| IL2 | A | - | - | 0 | 4 | 11 | 6 | 3 | 24 |
| IL3 | - | A | A | 0 | 4 | 1 | 2 | 2 | 9 |
| IL4 | - | - | A | 7 | 1 | 0 | 1 | 0 | 9 |
|  |  |  | total: | 16 | 16 | 16 | 16 | 14 | 78 |

| MADH3 | Positions | | | | | Counts | | | | | |
| --- | --- | --- | --- | --- | --- | --- | --- | --- | --- | --- | --- |
|  | -1946 | -1938 | -1137 | -479 | -669 | AFR | AME | ASI | EUR | MEA | TOT |
| **HapName** |  |  |  |  |  |  |  |  |  |  |  |
| **Ancestral** | **C** | **T** | **T** | **G** | **C** |  |  |  |  |  |  |
|  |  |  |  |  |  |  |  |  |  |  |  |
| MAD1 | - | - | - | - | - | 17 | 16 | 14 | 15 | 11 | 73 |
| MAD3 | - | - | - | A | - | 1 | 7 | 2 | 6 | 5 | 21 |
| MAD4 |  | C | - | - | T | 9 | 4 | 4 | 6 | 8 | 31 |
| MAD5 | - | C | G | - | T |  |  | 2 |  |  | 2 |
| MAD6 | A | - | - | - |  | 5 | 3 | 10 | 5 | 8 | 31 |
|  |  |  |  |  | total: | 32 | 30 | 32 | 32 | 32 | 158 |

| MADH4 | Positions | | Counts | | | | | |
| --- | --- | --- | --- | --- | --- | --- | --- | --- |
|  | -418 | -412 | AFR | AME | ASI | EUR | MEA | TOT |
| **HapName** | - | - |  |  |  |  |  |  |
| **Ancestral** | **A** | **G** |  |  |  |  |  |  |
| MADH4-01 | - | - | 28 | 32 | 30 | 32 | 32 | 154 |
| MADH4-02 | C | - | 3 |  |  |  |  | 3 |
| MADH4-03 | C | T | 1 | 0 | 0 | 0 | 0 | 1 |
|  |  | total: | 32 | 32 | 30 | 32 | 32 | 158 |

| MICA | Positions | | | | | | | | | | | | | | | Counts | | | | | |
| --- | --- | --- | --- | --- | --- | --- | --- | --- | --- | --- | --- | --- | --- | --- | --- | --- | --- | --- | --- | --- | --- |
|  | -1709 | -1483 | -1364 | -1224 | -1004 | -708 | -649 | -418 | -366 | -326 | -284 | -270 | -29 | -16 | 60 | AFR | AME | ASI | EUR | MEA | TOT |
| **HapName** |  |  |  |  |  |  |  |  |  |  |  |  |  |  |  |  |  |  |  |  |  |
| **Ancestral** | **G** | **G** | **A** | **A** | **G** | **C** | **C** | **C** | **G** | **G** | **C** | **G** | **C** | **G** | **C** |  |  |  |  |  |  |
| MIC01 | - | - | - | - | - | - | - | - | - | - | - | - | - | - | - | 3 | 4 | 5 | 6 | 4 | 22 |
| MIC02 | - | - | - | - | - | - | - | - | - | - | - | C | - | - | - | 2 | 5 | 0 | 4 | 4 | 15 |
| MIC03 | - | - | - | - | - | - | - | - | - | - | - | - | - | - | T | 2 | 1 | 1 | 2 | 2 | 8 |
| MIC04 | - | - | C | - | - | - | - | - | - | - | - | - | - | - | - | 2 | 1 | 0 | 0 | 0 | 3 |
| MIC05 | - | - | - | - | - | - | - | - | - | A | - | - | - | - | T | 0 | 0 | 2 | 1 | 1 | 4 |
| MIC06 | - | - | - | - | A | - | - | - | - | - | - | - | - | - | - | 0 | 0 | 3 | 0 | 0 | 3 |
| MIC07 | - | - | - | - | - | T | - | - | - | - | - | - | - | - | - | 0 | 1 | 1 | 2 | 0 | 4 |
| MIC08 | - | - | - | - | - | - | - | - | - | - | - | C | G | - | - | 0 | 0 | 1 | 0 | 0 | 1 |
| MIC09 | - | - | - | - | - | - | - | - | - | C | - | - | - | - | - | 2 | 0 | 0 | 0 | 0 | 2 |
| MIC10 | A | - | - | - | - | - | - | - | - | - | - | C | - | - | - | 0 | 3 | 0 | 1 | 2 | 6 |
| MIC11 | - | - | - | - | - | - | T | - | - | - | - | - | - | - | - | 0 | 0 | 2 | 0 | 0 | 2 |
| MIC12 | A | - | - | - | - | - | T | - | - | - | - | - | - | A | - | 1 | 0 | 0 | 0 | 1 | 2 |
| MIC13 | - | T | C | - | - | - | - | - | - | - | - | - | - | - | - | 1 | 0 | 0 | 0 | 0 | 1 |
| MIC14 | - | - | - | - | - | - | - | - | - | - | T | - | - | - | - | 2 | 0 | 0 | 0 | 0 | 2 |
| MIC15 | - | - | - | C | - | - | - | - | - | - | - | - | - | - | - | 0 | 1 | 0 | 0 | 0 | 1 |
| MIC16 | - | - | - | - | - | - | - | T | - | - | - | - | - | - | - | 0 | 0 | 1 | 0 | 0 | 1 |
| MIC17 | - | - | - | - | - | - | - | - | C | - | - | - | - | - | - | 1 | 0 | 0 | 0 | 0 | 1 |
|  |  |  |  |  |  |  |  |  |  |  |  |  |  |  | total: | 16 | 16 | 16 | 16 | 14 | 78 |

| RB1 | Positions | | | | | Counts | | | | | |
| --- | --- | --- | --- | --- | --- | --- | --- | --- | --- | --- | --- |
| **HapName** | -1733 | -1554 | -768 | -595 | -83 | AFR | AME | ASI | EUR | MEA | TOT |
| **Ancestral** | **-** | **C** | **G** | **A** | **INS a** |  |  |  |  |  |  |
| RB1 | - | - | - | - | - | 8 | 12 | 12 | 13 | 14 | 59 |
| RB2 | - | - | - | - | DEL | 0 | 0 | 0 | 1 | 0 | 1 |
| RB3 | - | - | T | - | - | 4 | 0 | 0 | 0 | 0 | 4 |
| RB4 | - | A | - | - | - | 0 | 2 | 4 | 2 | 2 | 10 |
| RB5 | - | A | - | C | - | 0 | 2 | 0 | 0 | 0 | 2 |
| RB6 | INS b | - | - | - | - | 4 | 0 | 0 | 0 | 0 | 4 |
| a) INS a = CTCGCGGACGTGACGCCGCGGGC | | | | | total: | 16 | 16 | 16 | 16 | 16 | 80 |
| b) INS b = INS CTGATA | | | | |  |  |  |  |  |  |  |

| SKP2 | Positions | | Counts | | | | | |
| --- | --- | --- | --- | --- | --- | --- | --- | --- |
|  | -846 | -758 | AFR | AME | ASI | EUR | MEA | TOT |
| **HapName** |  |  |  |  |  |  |  |  |
| **Ancestral** | **A** | **G** |  |  |  |  |  |  |
| SKP2-01 | - | - | 14 | 15 | 13 | 16 | 16 | 74 |
| SKP2-02 | - | C | 2222 | 0 | 0 | 0 | 0 | 2 |
| SKP2-03 | G | - |  | 1 | 1 | 0 | 0 | 2 |
|  |  | total: | 16 | 16 | 14 | 16 | 16 | 78 |

| TFDP1 | Positions | | | | | | | | Counts | | | | | |
| --- | --- | --- | --- | --- | --- | --- | --- | --- | --- | --- | --- | --- | --- | --- |
|  | -1887 | -1883 | -1779 | -1655 | -1530 | -1516 | -1515 | -1394 | AFR | AME | ASI | EUR | MEA | TOT |
| **HapName** |  |  |  |  |  |  |  |  |  |  |  |  |  |  |
| **Ancestral** | **C** | **G** | **A** | **G** | **C** | **C** | **G** | **T** |  |  |  |  |  |  |
| TFDP1-01 | - | - | - | - | - | - | - | - | 19 | 32 | 23 | 32 | 30 | 136 |
| TFDP1-02 | - | - | - | - | T | T | T | - | 4 | 0 | 4 | 0 | 0 | 8 |
| TFDP1-03 | - | - | G | - | - | - | - | - | 0 | 0 | 1 | 0 | 2 | 3 |
| TFDP1-05 | T | C | - | - | - | - | - | - | 1 |  |  |  |  | 1 |
| TFDP1-06 | T | C | - | A | - | - | - | C | 6 | 0 | 2 | 0 | 0 | 8 |
|  |  |  |  |  |  |  |  | total: | 30 | 32 | 30 | 32 | 32 | 156 |

| TGFB1 | Positions | | | | | | | | | Counts | | | | | |
| --- | --- | --- | --- | --- | --- | --- | --- | --- | --- | --- | --- | --- | --- | --- | --- |
|  | -1960 | -1886 | -1571 | -1550 | -1349 | -1146 | -799 | -508 | -448 | AFR | AME | ASI | EUR | MEA | TOT |
| **HapName** |  |  |  |  |  |  |  |  |  |  |  |  |  |  |  |
| **Ancestral** | **G** | **C** | **T** | **CCT** | **-** | **G** | **C** | **G** | **C** |  |  |  |  |  |  |
| TG1 | - | - | - | DEL | - | - | - | A | - | 2 | 8 | 9 | 5 | 8 | 32 |
| TG3 | - | - | - | DEL | - | C | - | A | - | 1 | 0 | 0 | 0 | 0 | 1 |
| TG4 | - | - | - | DEL | INS C | - | - | A | - | 0 | 1 | 0 | 0 | 0 | 1 |
| TG5 | - | - | - | - | - | - | - | A | - | 1 | 0 | 0 | 0 | 0 | 1 |
| TG8 | - | T | - | - | - | - | - | - | - | 0 | 1 | 0 | 0 | 2 | 3 |
| TG9 | - | T | C | - | - | - | - | - | - | 7 | 6 | 7 | 10 | 6 | 36 |
| TG10 | - | T | C | - | - | - | T | - | - | 0 | 0 | 0 | 1 | 0 | 1 |
| TG11 | A | - | - | - | - | - | - | - | - | 2 | 0 | 0 | 0 | 0 | 2 |
| TG12 | A | - | - | - | - | - | - | - | T | 1 | 0 | 0 | 0 | 0 | 1 |
|  |  |  |  |  |  |  |  |  | total: | 14 | 16 | 16 | 16 | 16 | 78 |

**Supplementary Table S4: Individual polymorphic sites with Fst values greater than two standard deviations above the average based on the empirical distribution in Suppl. Fig. 3S presenting Fst values of all 254 polymorphisms analyzed here**

| **Gene segment** | **Polymorphic site** | **Total sample** | | **Non-African sample** | |
| --- | --- | --- | --- | --- | --- |
|  |  | **New allele frequency** | **F_st_ (%) *** | **New allele frequency** | **F_st_ (%) *** |
| **CDKN1B** | rs36228498: T > G | 0.23 | 16.3 | 0.17 | 13.2 |
|  | rs3759217: C > T | 0.12 | (10.6) | 0.12 | 15.1 |
| **CX3CR1** | rs2669846: G > T | 0.40 | (11.0) | 0.41 | 14.6 |
|  | rs11715522: A > C | 0.43 | 19.4 | 0.55 | (8.4) |
|  | rs11917223 : C > G | 0.34 | 25.3 | 0.42 | 15.8 |
| **FEN1** | rs174538: G > A | 0.39 | 21.0 | 0.47 | (10.4) |
| **GPX2** | rs2296327: T > C | 0.59 | 20.6 | 0.64 | 24.7 |
|  | rs3831077: T > del T | 0.37 | 23.8 | 0.33 | 27.7 |
|  | rs17093568: C > A | 0.20 | 26.4 | 0.22 | 31.3 |
| **GPX3** | rs3763013: C > T | 0.62 | 21.5 | 0.72 | (0.7) |
| **GSTP1** | rs8191437: G > C | 0.47 | 17.1 | 0.55 | (7.7) |
| **HDAC1** | rs36212116: G > A | 0.95 | 22.6 | 1.00 | - |
|  | rs1741981: C > T | 0.49 | 26.4 | 0.60 | (3.0) |
| **IL1A** | rs3783521: G > A | 0.37 | 24.2 | 0.49 | (8.3) |

* F_st_ values that are below the threshold (average + 2 SD) are shown between parentheses.

**SUPPLEMENTARY FIGURES**

**Supplementary Figure S1:** Haplotype networks in alphabetical order with segment names as listed in Table 1 from the main document (can be found in Table 1S as well). The area of the circle is proportional to the overall haplotype frequency, with the same scale used across all loci, while colors indicate the redistribution among continental groups (red-Africa; yellow-South East Asian; green-America; brown-Middle-East; light blue-Europe). Haplotype networks without recombinations, where all haplotypes are reconnected by solid lines representing mutations, correspond to the maximum parsimony trees. In segments with recombination dashed lines represent sites with “recurrent” mutations always, which imitate the effect of recombinations and/ or gene conversions. In the presence of a single crossover (e.g CAT, CCND1, GSTM3 and MICA) plausible recombination partners are proposed and incorporated into the network. In complex cases possible recombination pathways are represented as composite reticulations and/or indicated as recurrent mutations at outside network branches. Note that the reconnecting lines representing the “recurrent” sites are always parallel. Reticulations either suggest plausible recombinations or indicate different possibilities in complex networks where a probable sequence of events cannot be deduced.

**
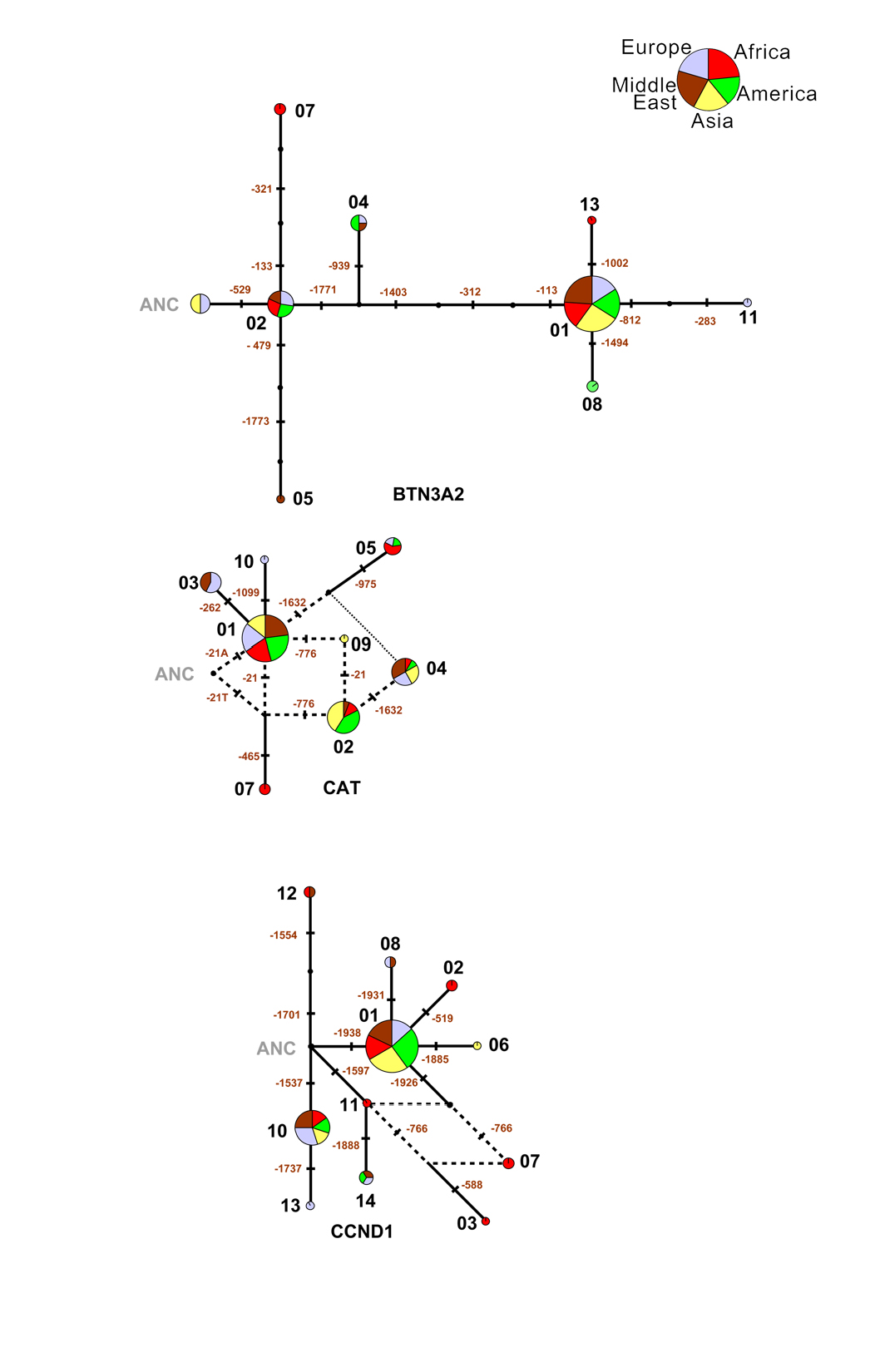
**

**
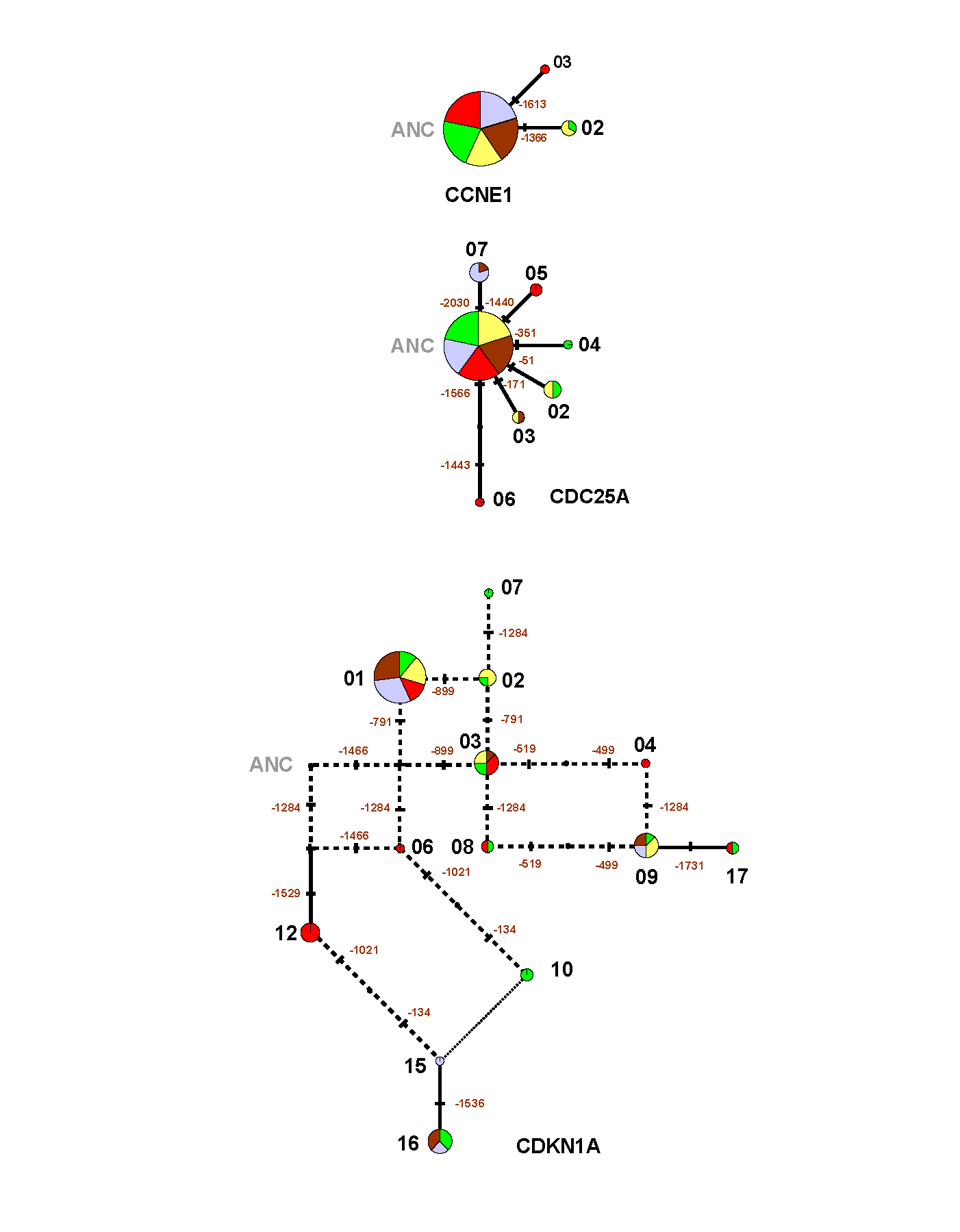
**

**
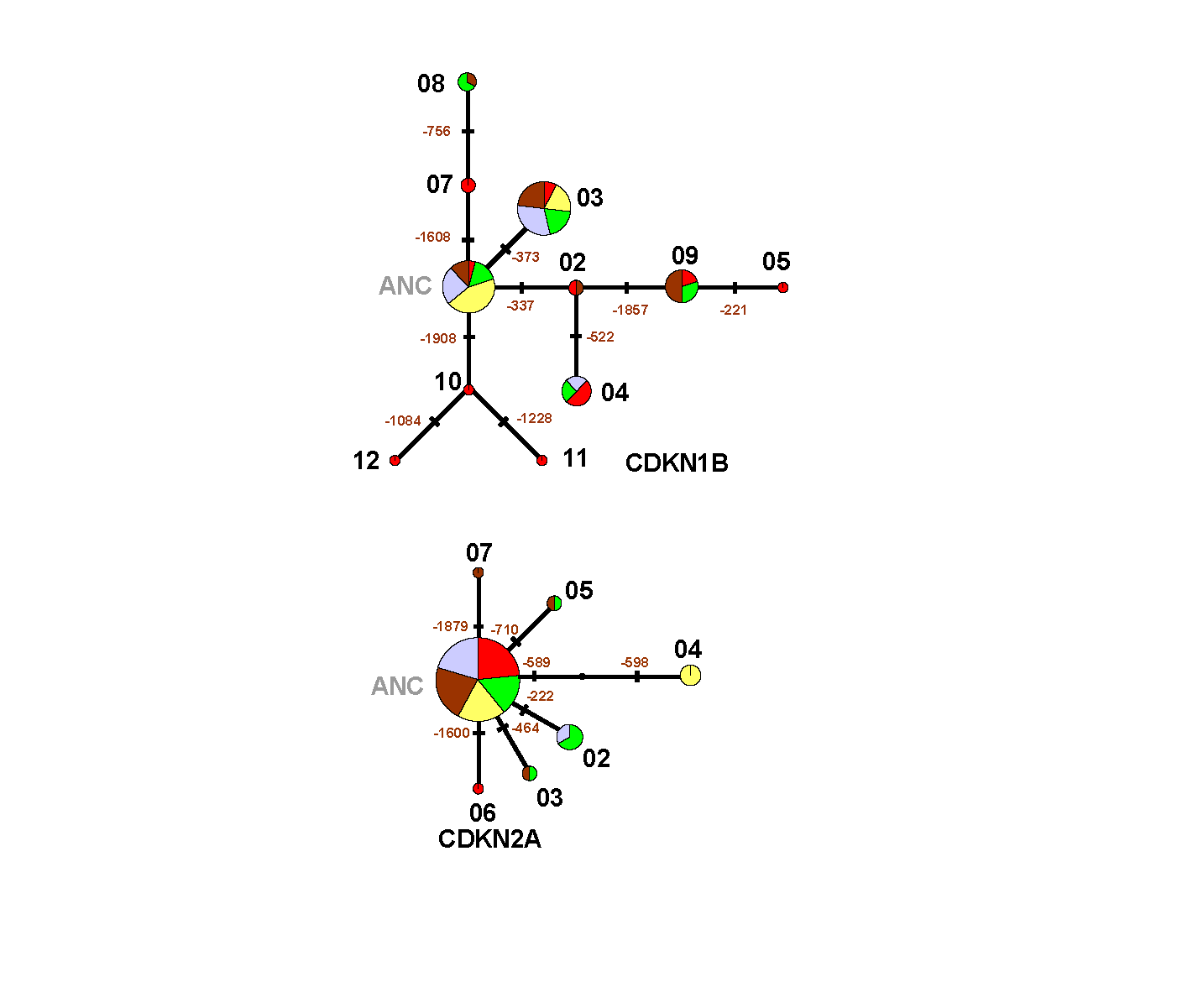
**

**
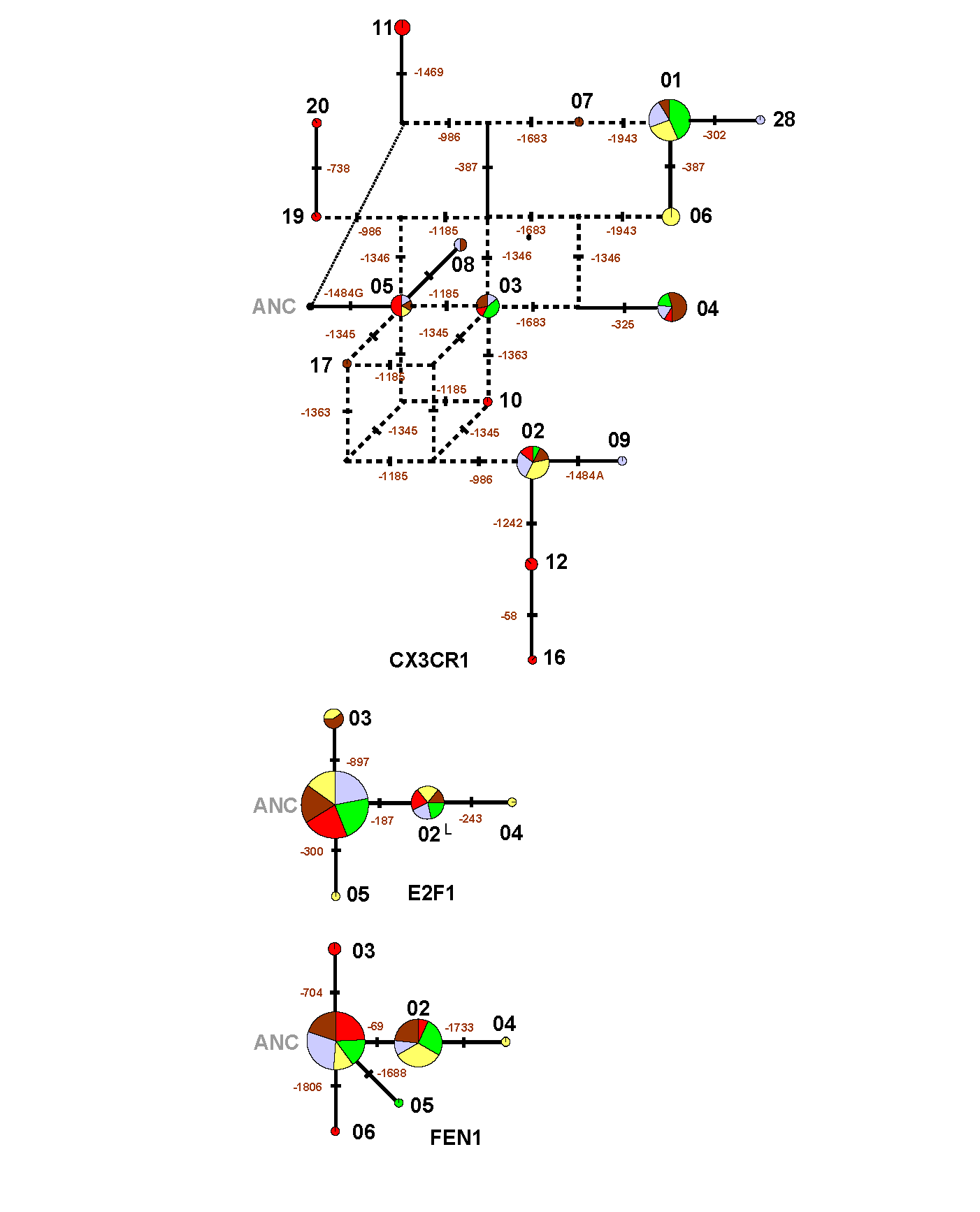
**

**
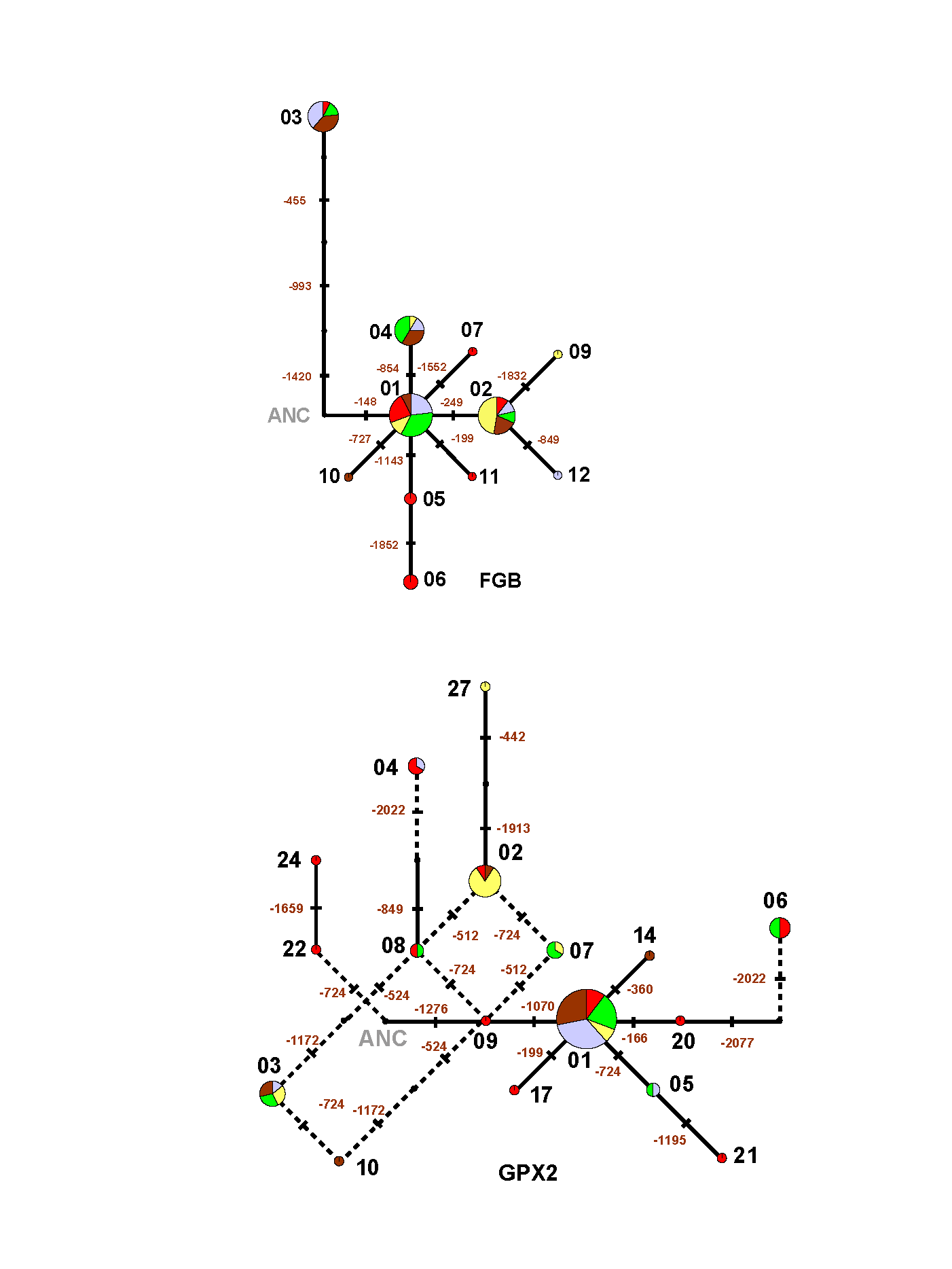
**

**
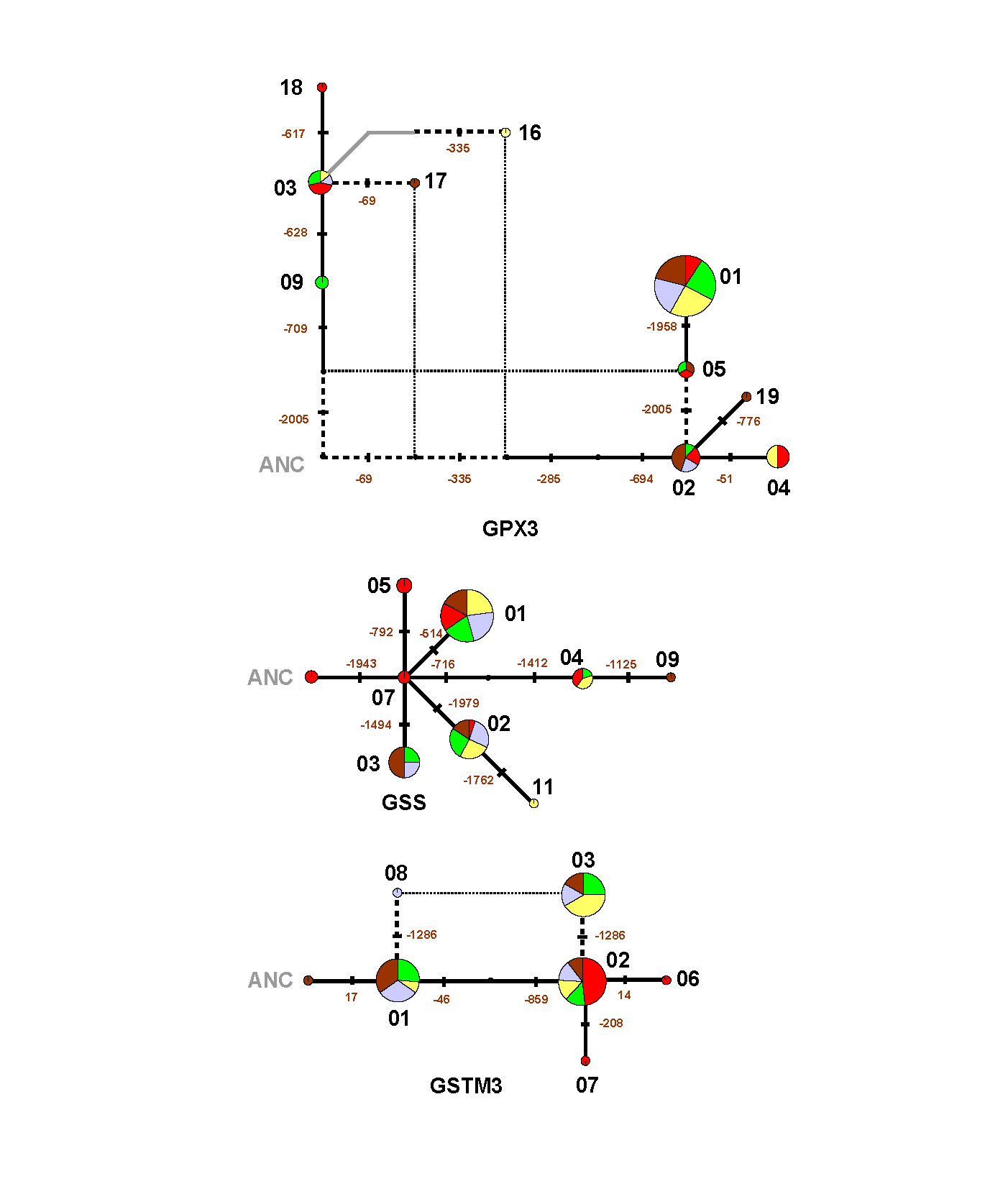
**

**
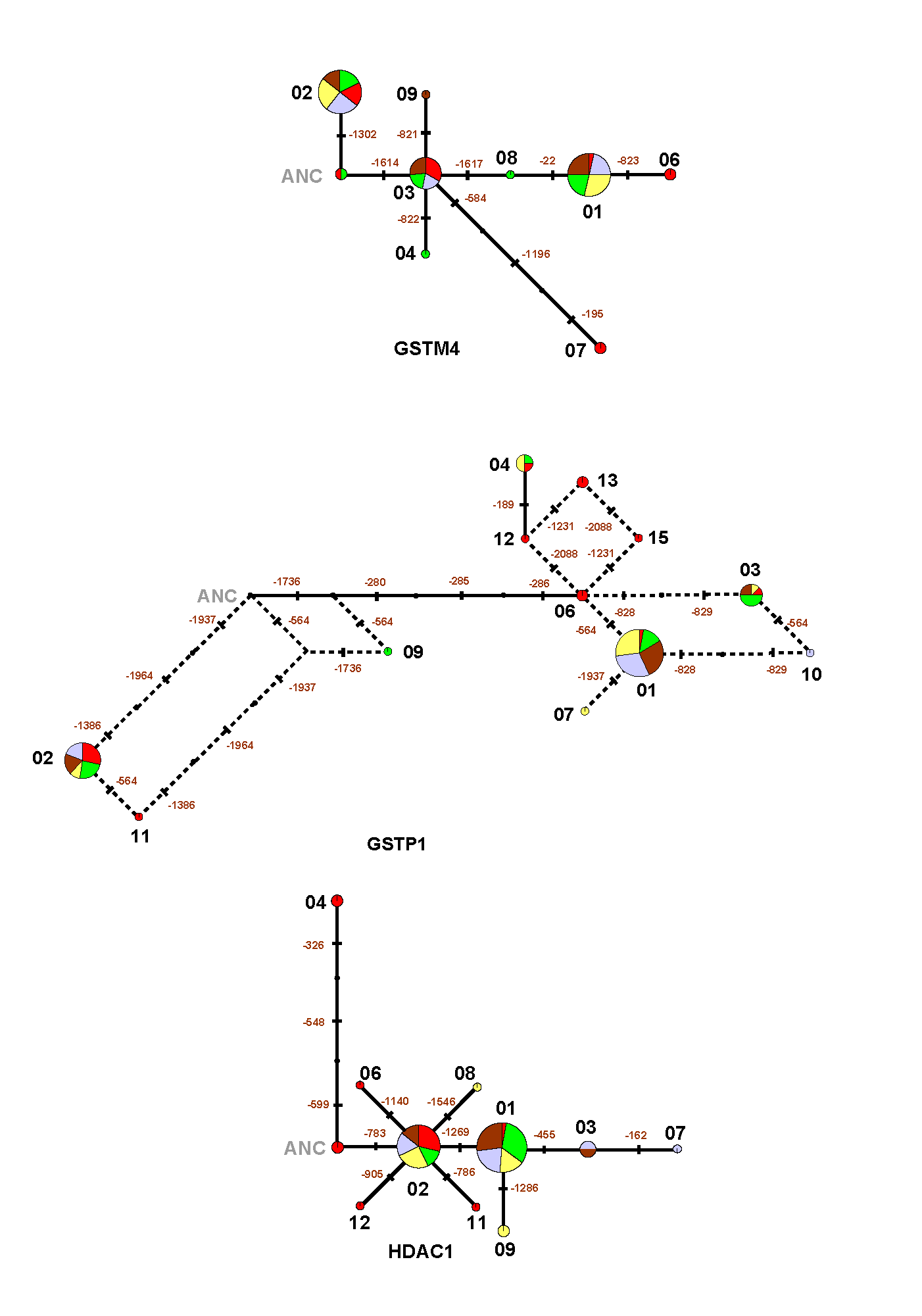
**

**
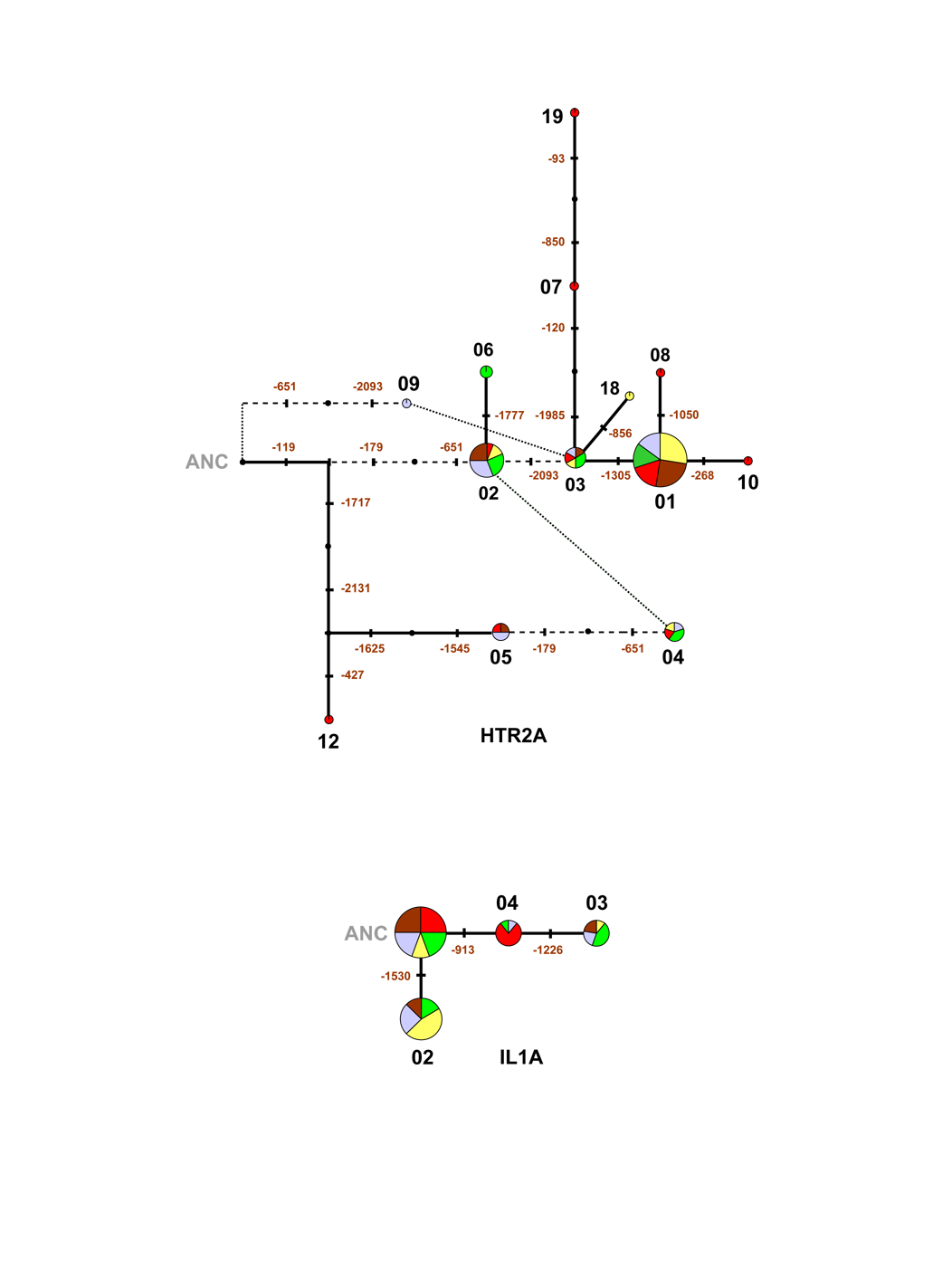
**

**
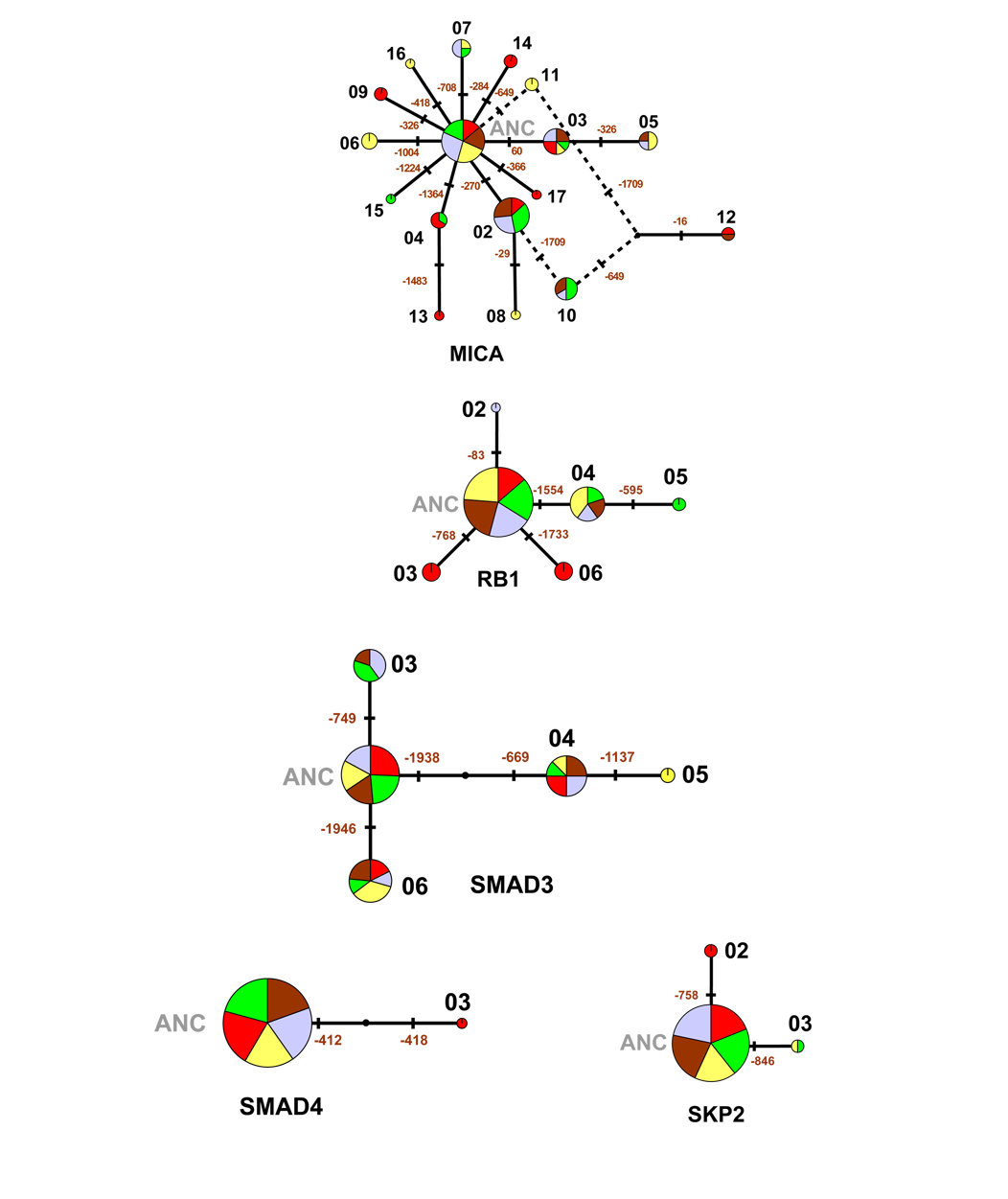
**

**
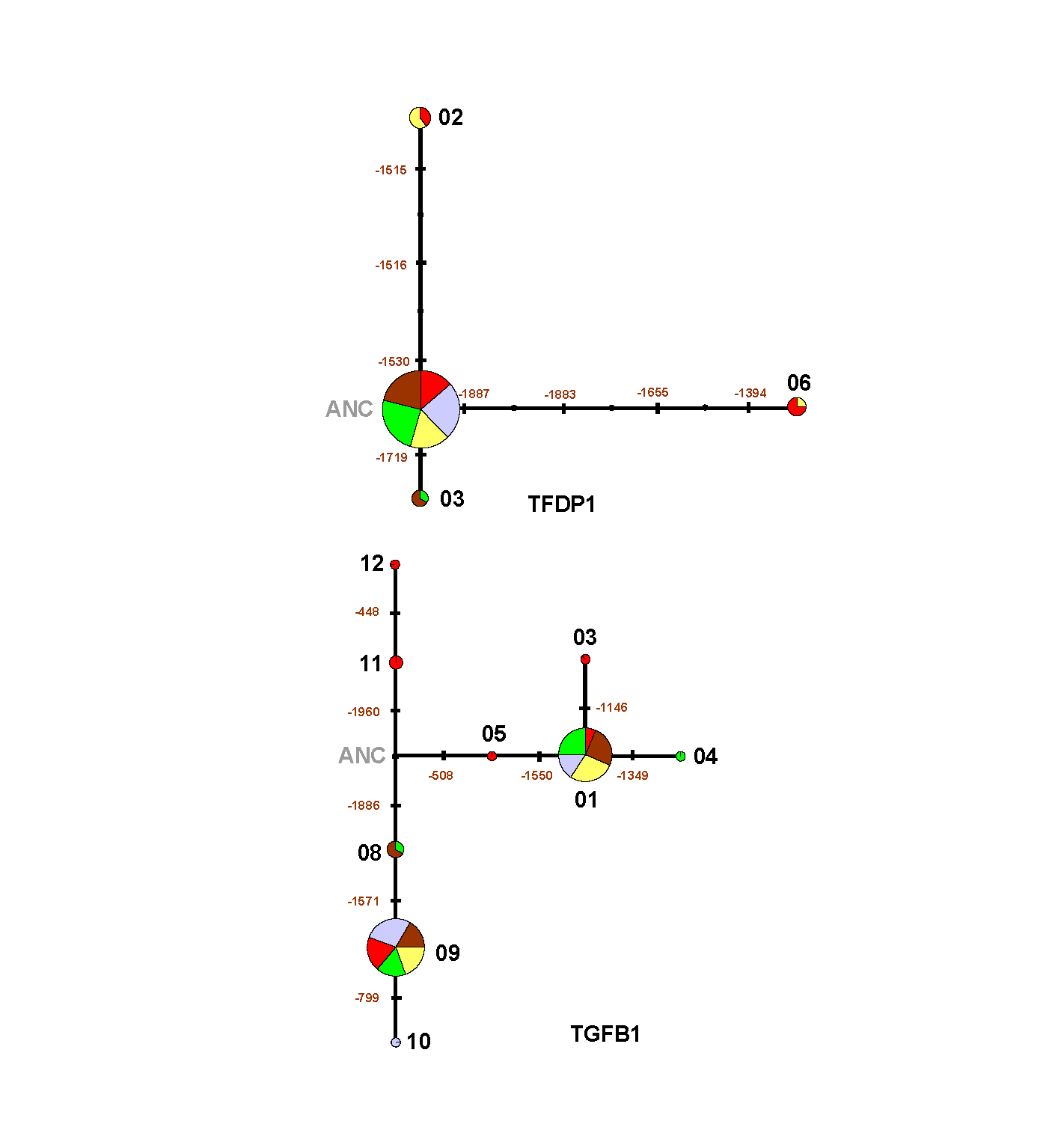
**

**Supplementary Figure S2:** Distributions of allelic frequency classes (left panels) of frequencies of haplotypes (middle) and haplotype allelic classes (right) in eighteen segments highlighted by neutrality tests and Fst measures. Bars represent the observed values and lines theoretical distributions. The occupancy of allelic frequency classes corresponds to counts of sites represented by *i* new alleles in a sample of *n* chromosomes (*i* = 1, 2, 3…n-1). Here, the theoretical curve (solid line) corresponds to the distribution calculated form the equation (Fu 1997, Fan et al 2002) S*_i_* (*i*) = Θ/*i*, using Θ/*_Π_* (Table 1 from the main document) as the estimator of Θ. The theoretical distribution (solid line) of haplotype frequencies expected given *k* observed haplotypes (Table 1 from the main document) is according to Ewens (1972). Haplotype names are arbitrary and correspond to their names in our database. In the case of haplotype allelic classes, regrouping haplotypes sharing the same number of mutations from the ancestral haplotype, their theoretical occupancy was obtained by coalescent simulation under standard model assuming constant population size without (solid line) and with recombination (dotted line), tenfold the genomic average in the case of segments where crossovers were detected.


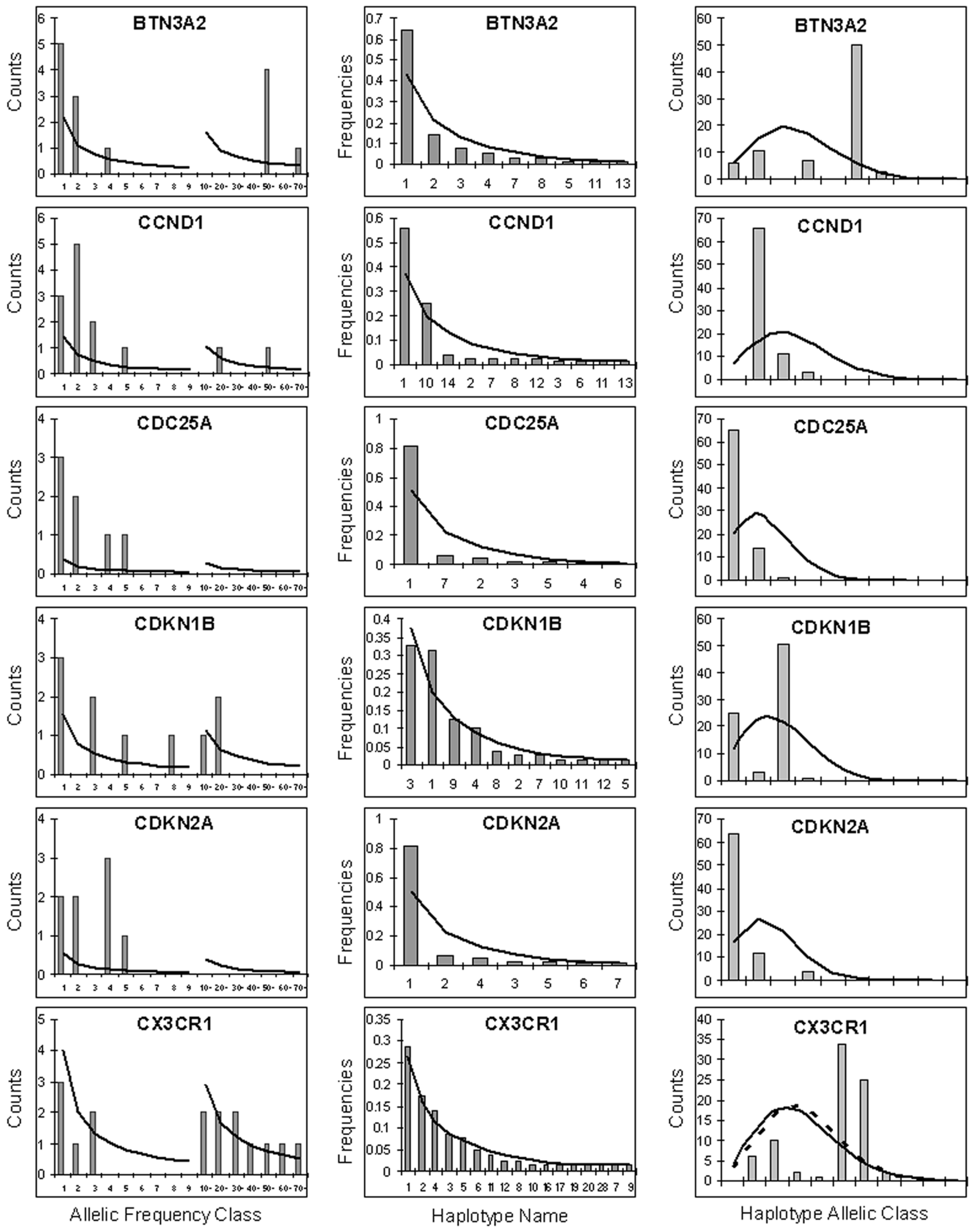


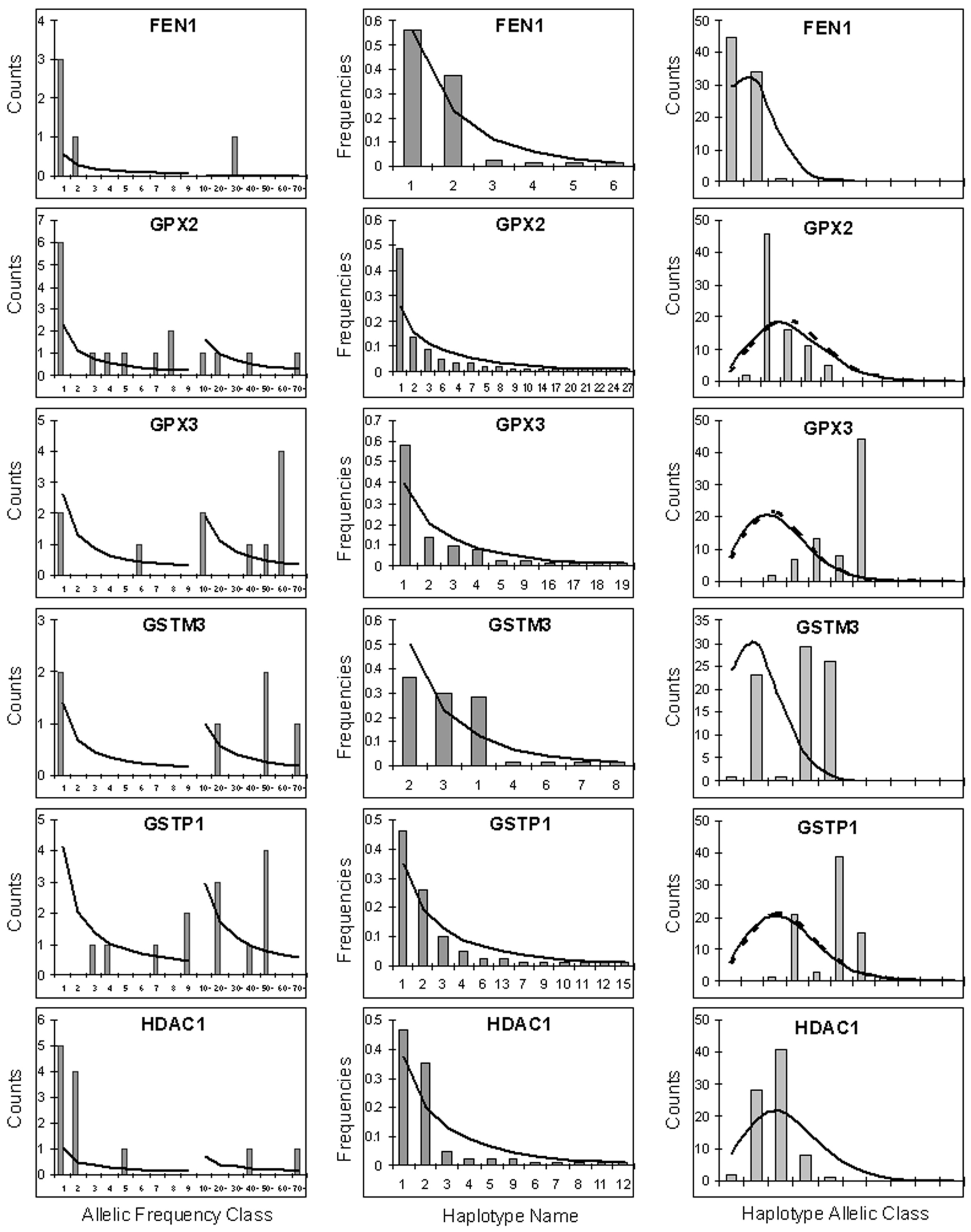


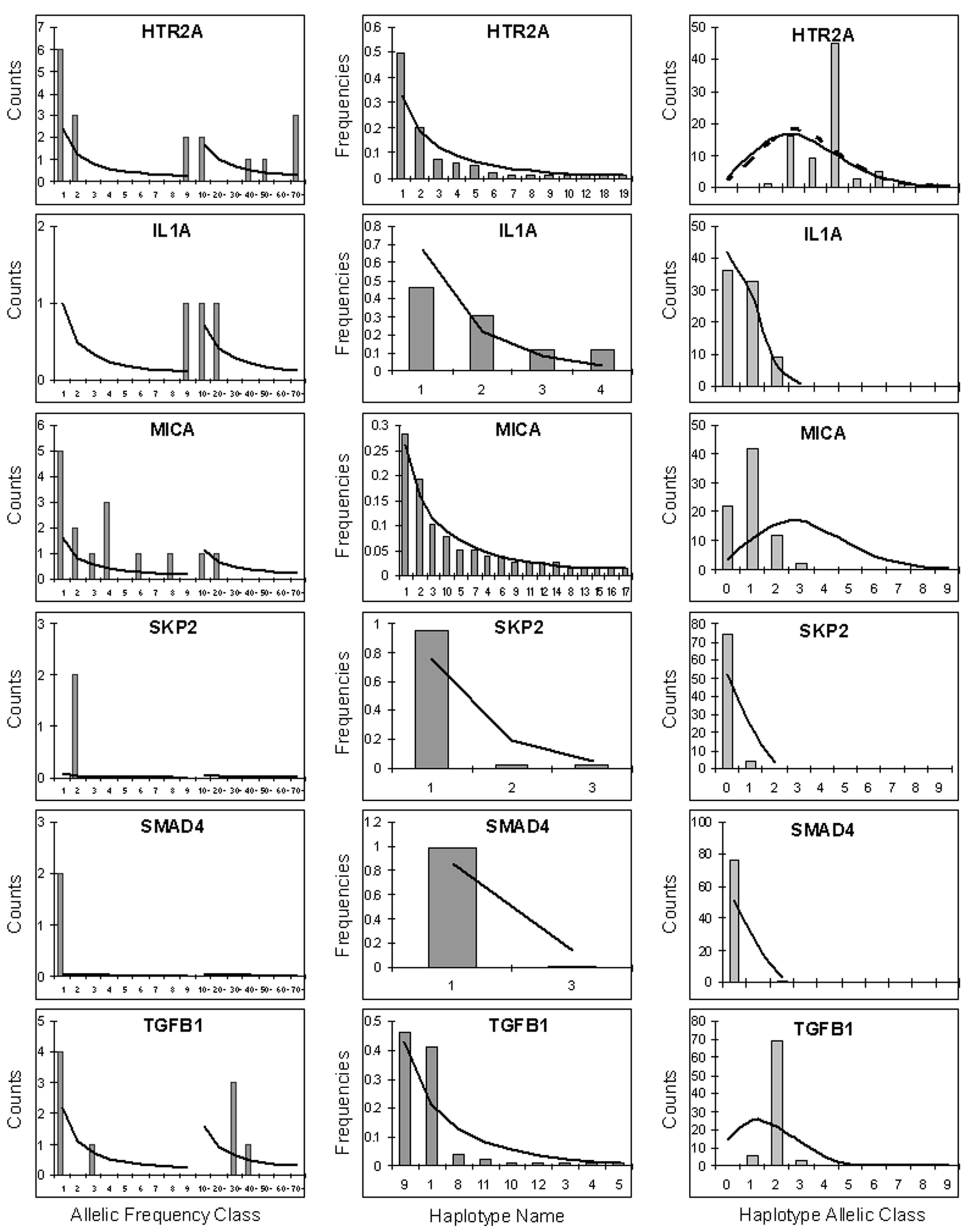


**Supplementary Figure S3:** Distribution of Fst values (Weir and Cockerham 1984) per polymorphic site, rank ordered from the smallest to highest values, in the total sample (World), represented by five population groups (254 sites) in A, and in the four population sample with exclusion of SubSaharan Africans (191 sites) in B. Negative Fst values were put to zero. These distributions are compared to these obtained in the same way using data set on 297 sites from Fan et al. (2002), for the World (C) composed of 4 population groups of African, Amerindian, East Asiatic and European descent (8 individuals each) and for the non-African composed of three non-African groups above (D).

.

**
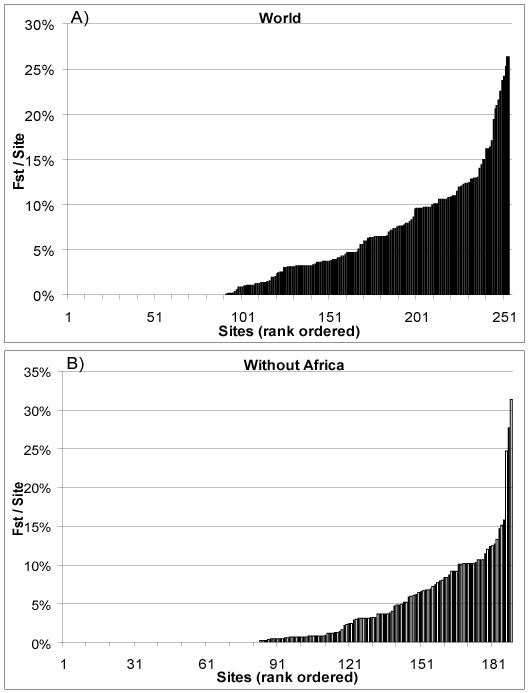
**


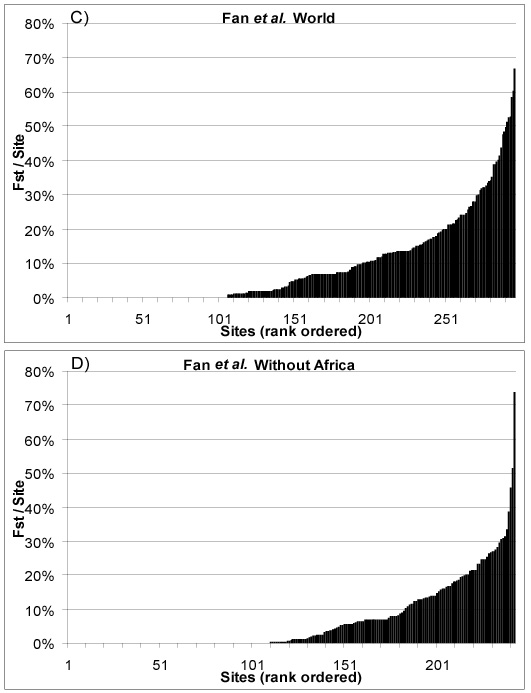
Distribution of Fst values (Weir and Cockerham 1984) per polymorphic site, rank ordered from the smallest to highest values, in the total sample (World), represented by five population groups (254 sites) in A, and in the four population sample with exclusion of SubSaharan Africans (191 sites) in B. Negative Fst values were put to zero. These distributions are compared to these obtained in the same way using data set on 297 sites from Fan et al. (2002), for the World (C) composed of 4 population groups of African, Amerindian, East Asiatic and European descent (8 individuals each) and for the non-African composed of three non-African groups above (D).

**Supplementary Figure S4:** Frequencies of the ancestral haplotypes (left) and the distribution of major haplotypes among haplotype allelic classes (right) in 28 studied segments. The data (solid bars) are compared with theoretical expectation from coalescent simulations under the standard model in the absence of recombination (solid line) and assuming bottleneck (upper panels) and exponential growth (lower panels) as in Akey et al. 2004. Simulations were for sample size of 80 chromosomes, mutation rate of 2.13 x 10^-8^ per bp per generation, corresponding to the average S density of 9.1, and *N* = 10 000.

**
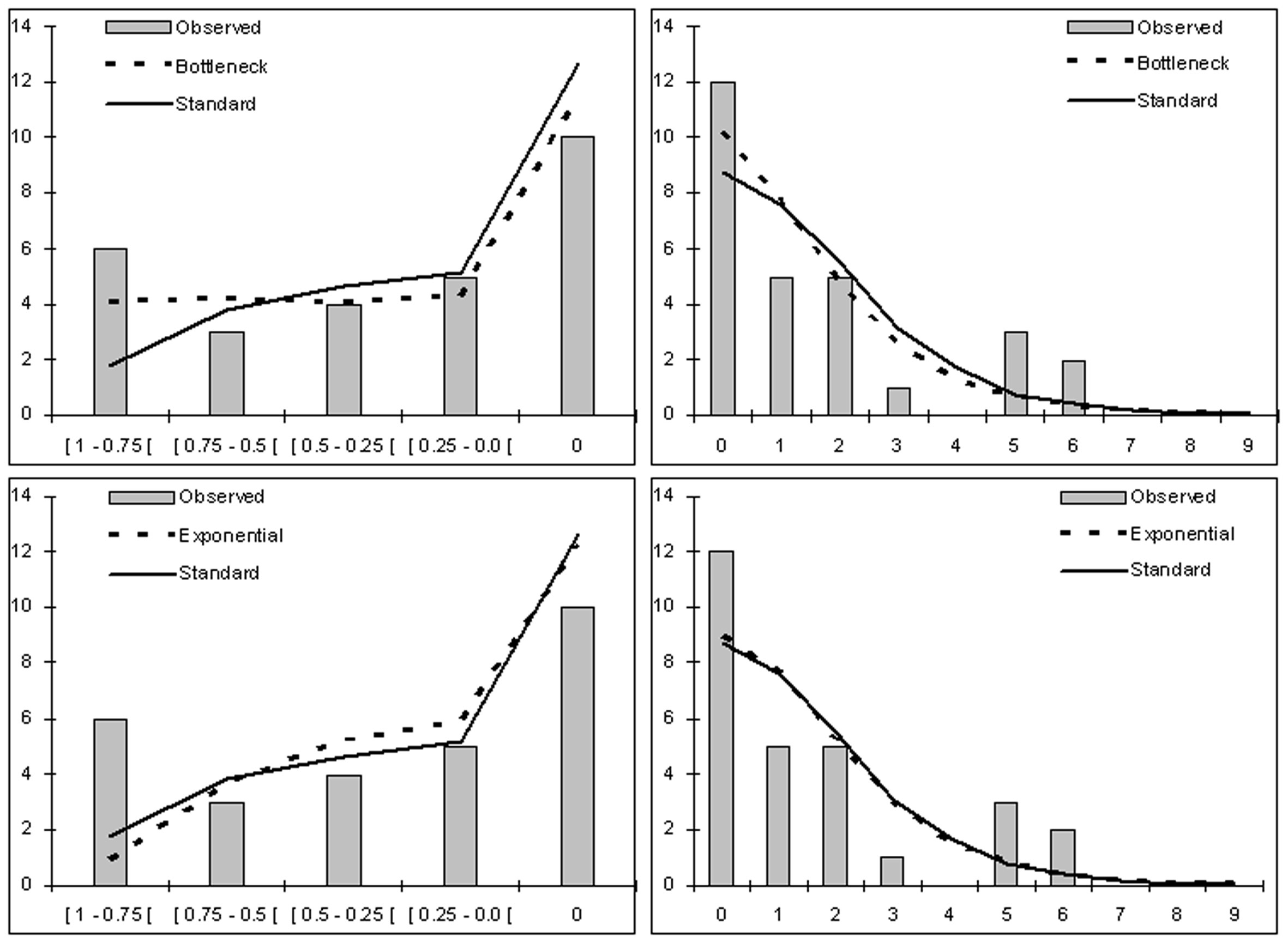
**
